# Human resource shortage in India’s health sector: a scoping review of the current landscape

**DOI:** 10.1186/s12889-024-18850-x

**Published:** 2024-05-21

**Authors:** Vini Mehta, Puneeta Ajmera, Sheetal Kalra, Mohammad Miraj, Ruchika Gallani, Riyaz Ahamed Shaik, Hashem Abu Serhan, Ranjit Sah

**Affiliations:** 1 Department of Dental Research Cell, Dr. D. Y. Patil Dental College and Hospital, Dr. D. Y. Patil Vidyapeeth, Pimpri, Pune, 411018 India; 2 Department of Public Health, School of Allied Health Sciences, Delhi Pharmaceutical Sciences and Research University, New Delhi, India; 3 School of Physiotherapy, Delhi Pharmaceutical Sciences and Research University, New Delhi, India; 4 Department of Physical Therapy and Health Rehabilitation, College of Applied Medical Sciences, Majmaah University, AlMajmaah, Saudi Arabia; 5 STAT SENSE, Gujarat, 382421 India; 6 Department of Family and Community Medicine, College of Medicine, Majmaah University, Al Majmaah, Saudi Arabia; 7 Department of Ophthalmology, Hamad Medical Corporation, Doha, Qatar; 8 SR Sanjeevani Hospital, Kalyanpur, Siraha Nepal

**Keywords:** Human resources for health, Health workforce, Shortage, India, South East Asia

## Abstract

**Background:**

For healthcare delivery to be optimally effective, health systems must possess adequate levels and we must ensure a fair distribution of human resources aimed at healthcare facilities. We conducted a scoping review to map the current state of human resources for health (HRH) in India and the reasons behind its shortage.

**Methods:**

A systematic search was conducted in various electronic databases, from the earliest available date till February 2024. We applied a uniform analytical framework to all the primary research reports and adopted the “descriptive-analytical” method from the narrative paradigm. Inductive thematic analysis was conducted to arrange the retrieved data into categories based on related themes after creating a chart of HRH problems.

**Results:**

A total of 9675 articles were retrieved for this review. 88 full texts were included for the final data analysis. The shortage was addressed in 30.6% studies (*n* = 27) whereas 69.3% of studies (*n* = 61) addressed reasons for the shortage. The thematic analysis of data regarding reasons for the shortage yielded five kinds of HRH-related problems such as inadequate HRH production, job dissatisfaction, brain drain, regulatory issues, and lack of training, monitoring, and evaluation that were causing a scarcity of HRH in India.

**Conclusion:**

There has been a persistent shortage and inequitable distribution of human resources in India with the rural expert cadres experiencing the most severe shortage. The health department needs to establish a productive recruitment system if long-term solutions are to be achieved. It is important to address the slow and sporadic nature of the recruitment system and the issue of job insecurity among medical officers, which in turn affects their other employment benefits, such as salary, pension, and recognition for the years of service.

**Supplementary Information:**

The online version contains supplementary material available at 10.1186/s12889-024-18850-x.

## Background

Universal healthcare is recognized as a basic human right by the World Health Organization (WHO). Human resources for health (HRH) are an essential component of effective and high-quality healthcare systems, which are responsible for the maintenance and promotion of good health. In order for health care delivery to be as effective as possible, health systems must have adequate levels and fair distribution of human resources for health [1, 2]. HRH are defined as “the stock of all individuals engaged in the promotion, protection or improvement of population health”. This includes both public and private sectors and different domains of health systems, such as personal curative and preventive care, non-personal public health interventions, disease prevention, health promotion services, research, management, and support services (WHO, 2007) [1]. The HRH is eventually required to carry out policies, conduct processes, prescribe medication, and offer care to the populace. Therefore, it should come as no surprise that nations with low physician densities are thought to do poorly in terms of life expectancy and maternal and child mortality [3, 4]. India is one of the 57 nations with a clear shortage of HRH [1, 6]. WHO recommends 44.5 doctors, nurses, and midwives per 10,000 inhabitants, whereas the national density was found to be 20.6 [7]. The current health worker density is noteworthy since it represents a significant improvement from the anticipated 13.6 per 10,000 in 2005 [8]. However, the distribution of HRH throughout the states is uneven [9, 10]. There are notable variances between urban and rural locations in HRH, with urban areas having a doctor density that is four times higher than rural ones. Availability, distribution, and quality of HRH are crucial for achieving universal health coverage (UHC) in lower-to-middle-income countries (LMICs) such as India. There have been multiple studies measuring the HRH shortage. There are also quantitative and qualitative studies looking at the reasons for the shortage. Here, we attempt to provide the most comprehensive scoping review of the estimates of the HRH shortage in India and a critical discussion of the reasons/factors underlying this shortage. To our knowledge, this would be the first review on the matter.

From a policy perspective, it is critical to comprehend how a country with a surplus of human resources structures its shortfall. Despite India’s obvious public health problems, the topic has received little attention from researchers. The academic literature on HRH in India from inception to January 2023 was reviewed here, along with the current state of affairs, trends, and the nature of the shortage. Therefore, this scoping review aims to map the current state of HRH in India and the reasons behind its shortage.

## Methods

This scoping review was conducted according to the Preferred Reporting Items for Systematic Reviews and Meta-Analysis: Extension for Scoping Reviews (PRISMA-SCR) [11]. A scoping review was most appropriate due to the broad nature of this subject and the range of study designs included. Furthermore, it was necessary to conduct a wide search encompassing studies that examined WHO-Sustainable Development Goals (SDGs) benchmarks, Indian Public Health Standards (IPHS) guidelines, and India-SDG benchmarks. On 2-11-2022 the completed protocol was prospectively registered with the Open Science Framework (10.17605/OSF.IO/6S4QB).

### Search strategy

An exhaustive literature search was conducted to identify the shortage and reasons for shortages of HRH in India. Online electronic databases such as PubMed-Medline, Embase, Scopus, Cochrane Library, Web of Science (WoS), the Cumulative Index to Nursing and Allied Health Literature (CINAHL), and EBSCO (Global Health) were searched from the earliest available date till February 2024. Additional sources like Google Scholar, WHO library database (WHOLIS), Public Health Foundation of India Knowledge Repository (PHFI), INDMED, conference proceedings, and cross-references were explored. Non-English language publications were translated into the English language using Google Translate [12]. Contact with authors was done for any unpublished studies. A detailed search strategy is given in Table 1 for PubMed-Medline and tailored to each database when necessary [Supplementary Table 1].

**Table 1:** Search strategy

Domains	Keywords
Human resources for health	(Human resources) OR (HRH) OR (health system) OR (Healthcare Planning) OR (health services) OR (Primary health care system) OR (Universal health coverage)
Health workforce	(Health workers) OR (Multi-purpose health workers) OR (Village Health Guides) OR (Health personnel) OR (Community health workers) OR (health associated workers) OR (Accredited Social Health Activist) OR (health care workers) OR (Health service providers) OR (Health worker shortage) OR (Community Health Workers) OR (“Health Personnel”) OR (“Health Workforce”) OR (Health Services)
Doctors	(Dentists) OR (Nurses) OR (Midwives) OR (Traditional & faith healers) OR (AYUSH) OR (Allopathic) OR (Dental auxiliaries) OR (Specialists) OR (Physicians) OR (Ayurvedic) OR (Homeopathy)
Shortage	shortage OR rural deployment OR medically underserved areas
Health	“Public Health”
India	India OR (Rural India) OR (South-East Asia)

### Eligibility criteria

We sought to define and characterize the state of shortage of HRH in India. In order to be included in the review, included studies needed to focus on metrics for shortage measurements such as density estimates, raw/absolute numbers, shortfall, and vacancies. We included studies that analyzed records from national, sub-national (state), district, administrative block, and center-level based on the comprehensive comprehensive list of cadres mentioned in the National Classification of Occupations (NCO) by the Government of India (GoI) [13], and the International Standard Classification of Occupations (ISCO-08) [14] by the International Labour Office was selected. Public, private, and public-private partnerships (PPPs), and social/non-governmental/trust were taken into consideration, making the list of cadres comprehensive. In the Indian healthcare industry, health workers are broadly classified as medical health professionals, including paramedical people and non-medical workers. The latter includes numerous categories of non-medical workers. They are classified as healthcare workers. They are classified as healthcare workers since they work in healthcare facilities.

### Screening and selection

We imported all search results into Zotero 5.0 and reimported all titles and abstracts into the Excel screening workbook. Two researchers independently screened (VM and RG), first by the title and abstract to verify the agreement between the reviewers on the inclusion and exclusion criteria. Case reports, letters, and narrative/historical reviews were not included in the search. The eligibility criteria were refined until a good agreement was reached. Papers without abstracts but with titles suggesting that they were related to the objectives of this review were also selected to screen the full text for eligibility. After selection, full‑text papers were read in detail by two reviewers (PA and SK). Those papers that fulfilled all of the selection criteria were processed for data extraction. Two reviewers (VM and RG) hand-searched the reference lists of all selected studies for additional relevant articles. The level of agreement between the two reviewers, calculated by Cohen’s kappa (k), was 0.92 for titles and abstracts and 0.90 for full texts. Disagreements between the two reviewers were resolved by discussion. If a disagreement persisted, the judgment of a third reviewer (MM) was considered decisive.

Also, studies examining HRH (absolute numbers/shortage/vacancy/shortfall) at national levels in urban and rural locations in India were considered for comparing the density of HRH. We carefully examined the papers to get information on HRH enumeration, openings, and deficits. For uniformity and comparison with WHO criteria, the available data was adjusted as necessary. For instance, all HRH densities are recalculated and given as 10,000 HRH workers.

### Data extraction and analysis

Two authors (VM and PA) independently extracted data using specially designed data extraction forms, utilizing Microsoft Excel software. Inter-rater reliability between the two authors was 0.8 for data extraction and analysis. The following informational data fields were used: author/year of publication, location, study design, sample size, study setting, study design, data collection tool, cadre shortages, career stages, employment status, reasons for the shortage, results, and conclusion of the studies. We applied descriptive analysis for objective one and thematic analysis approach for second objective. Since articles might belong to numerous categories, the total number of articles belonging to one category may be smaller than the total number of articles belonging to all other categories. In the text and the supplemental materials, figures depict the distributions of papers by publication year, journal, and therapeutic/practice area. Inductive thematic analysis, as defined by Braun and Clarke [15], was used to arrange the retrieved data into categories based on related themes. A thorough literature review was conducted and the following steps were undertaken to create a chart of HRH-related challenges in India:

1. Extensive literature search: For a thorough grasp of the major themes and topics that have surfaced, a comprehensive literature review was conducted.
2. Developing initial codes: Data was initially coded by determining the meaningful text units related to HRH shortages in India. The key descriptive and interpretative concepts and ideas contained in the data were captured by these codes.
3. Identify themes: After the initial codes were identified, connections and patterns among them were explored by the reviewers. Similar codes were grouped to generate five themes that reflected the underlying meanings and concepts in the data by utilizing an iterative and inductive method. Any disagreement was resolved by discussion between the authors.
4. Refine themes: The five themes were refined further in terms of wording and language and finally agreed upon by all the authors ensuring that all of them are coherent and accurately reflect the underlying meanings and ideas within the data.

The available data was modified for uniformity and comparison with WHO-SDG’s benchmarks, Indian Public Health Standards (IPHS) guidelines, and India-SDG benchmarks.

### Methodological quality appraisal

In line with guidelines for conducting a scoping review, no formal assessment of the methodological quality of the included articles was performed.

## Results

### Search and selection results

**Fig. 1:**
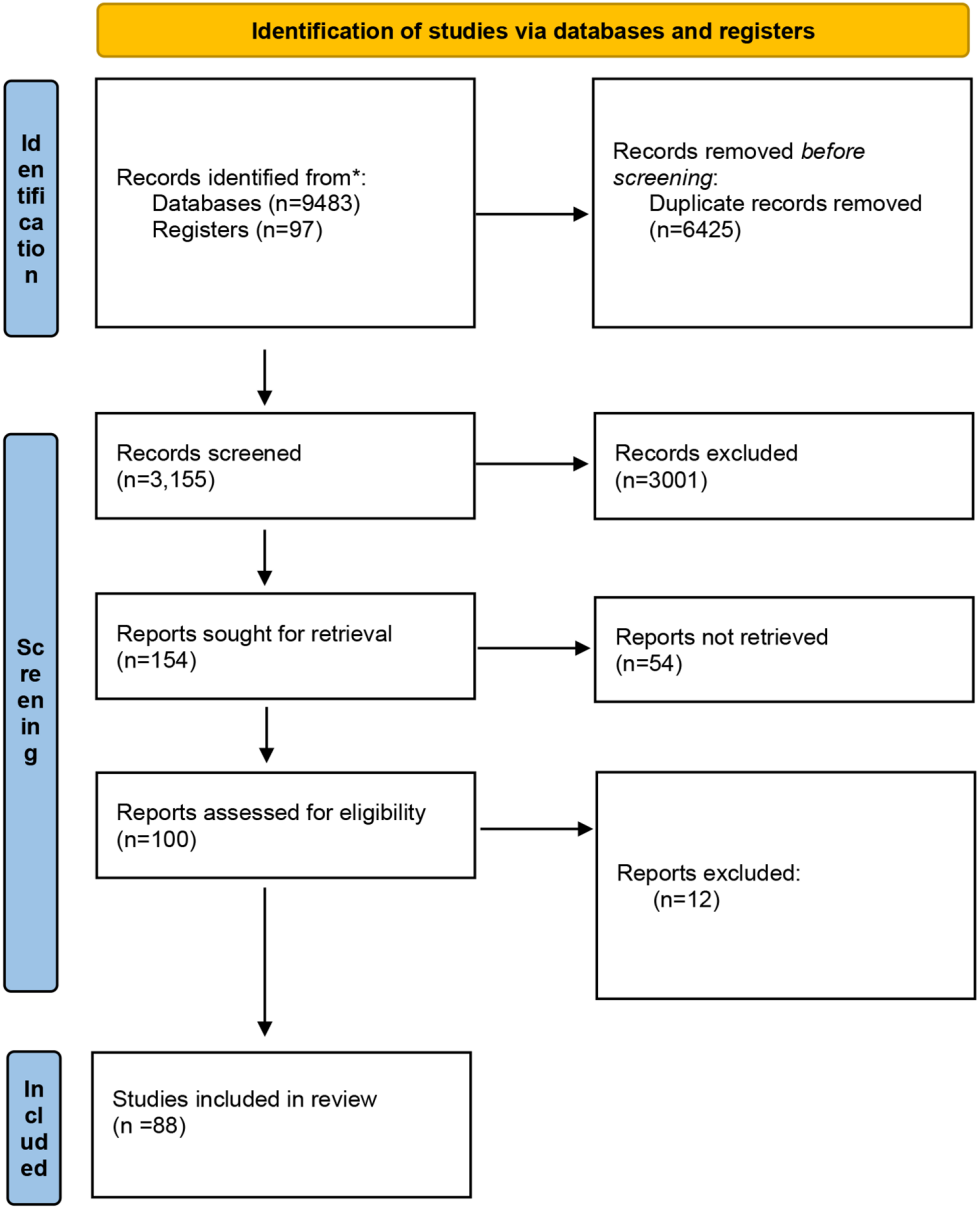
Flowchart summarizing the article selection process (*n* – number of studies)

**Fig. 2:**
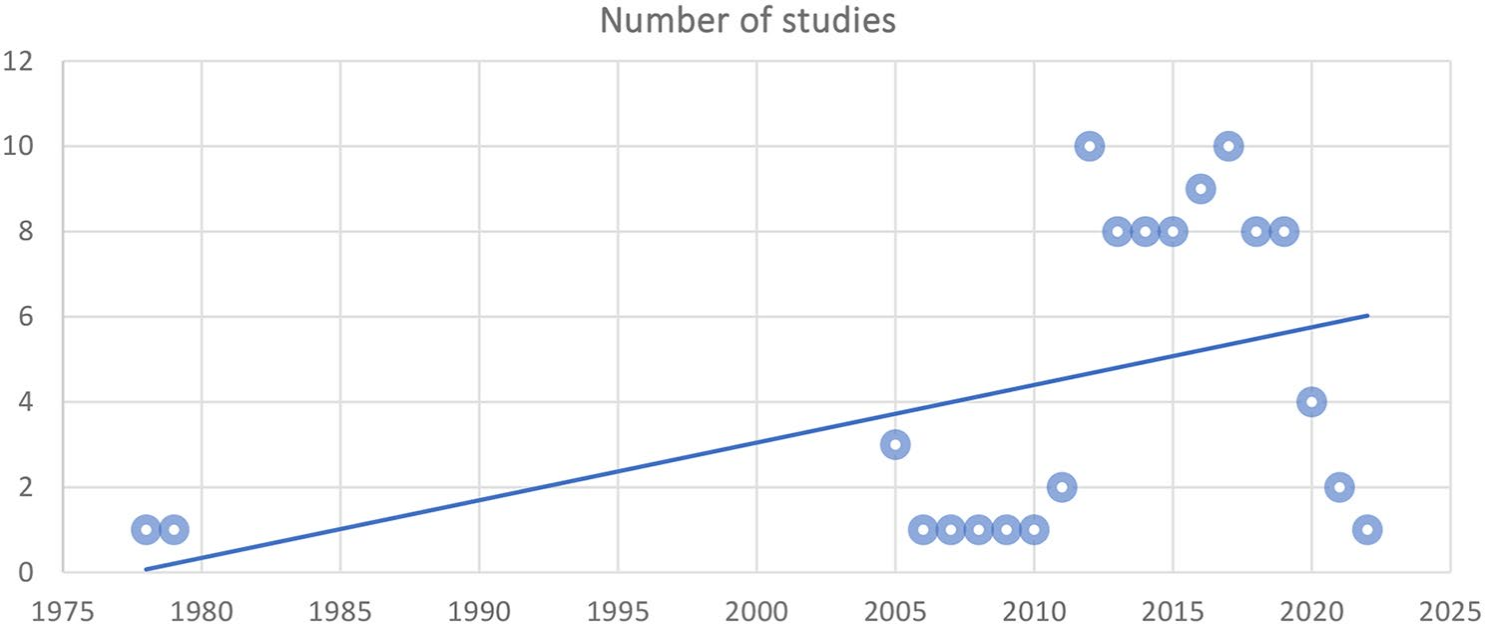
Number of studies according to publication years

A total of 9,580 articles were retrieved for this review, including 9483 from the databases and 97 from the additional sources. After removing duplicates, 3,155 articles remained for screening the titles. 154 articles were chosen for screening the abstracts, yielding 100 articles eligible for full-text screening. 88 full texts10,16–102 were included for the final data analysis (PRISMA flow diagram in Fig. 1). Study characteristics [10, 16–102] are described in detail in Supplementary Table 2. The first research question was addressed in 30.6% of studies (*n* = 27) whereas 69.3% of studies (*n* = 61) addressed the second research question. In Fig. 2, the publication years are displayed. The first article was published in 1978. 11.3% of articles (*n* = 10) were published before 2010 while 85.2% were published from 2011 to 2020. Highest number of papers (*n* = 10) were published in 2012 and 2017.

Articles were categorized into state level (HRH issues of only one state), national level (HRH issues of more than one state/multicentric), and international level (HRH issues of more than one country including India) for ease of understanding. 50% of studies (*n* = 44) were conducted at the state level focussing on the HRH of a single state. 36.3% of studies were multicentric (*n* = 32) and were conducted at the national level including more than one state of India while 13.6% (*n* = 12) were international level studies conducted in more than one country including India 67.7% of studies were based on primary data while 26.8% studies were based on secondary data obtained from different sources. In 5.3% of studies both primary as well as secondary data was used to collect data.

48.2% of studies were cross-sectional surveys. A questionnaire (*n* = 44, 89%) was used in the majority of the surveys for data collection. Qualitative methods such as interviews were also used in surveys, albeit less frequently (*n* = 16, 17.3%). One study used focus group discussion while in three studies, both interviews as well as focus group discussions were conducted. A mixed method study design (both qualitative and quantitative) was used in 6.4% of studies. In two qualitative studies, the Fujifilm Quick-Snap disposable camera was used to take photographs and conduct thematic analysis.

**Fig. 3:**
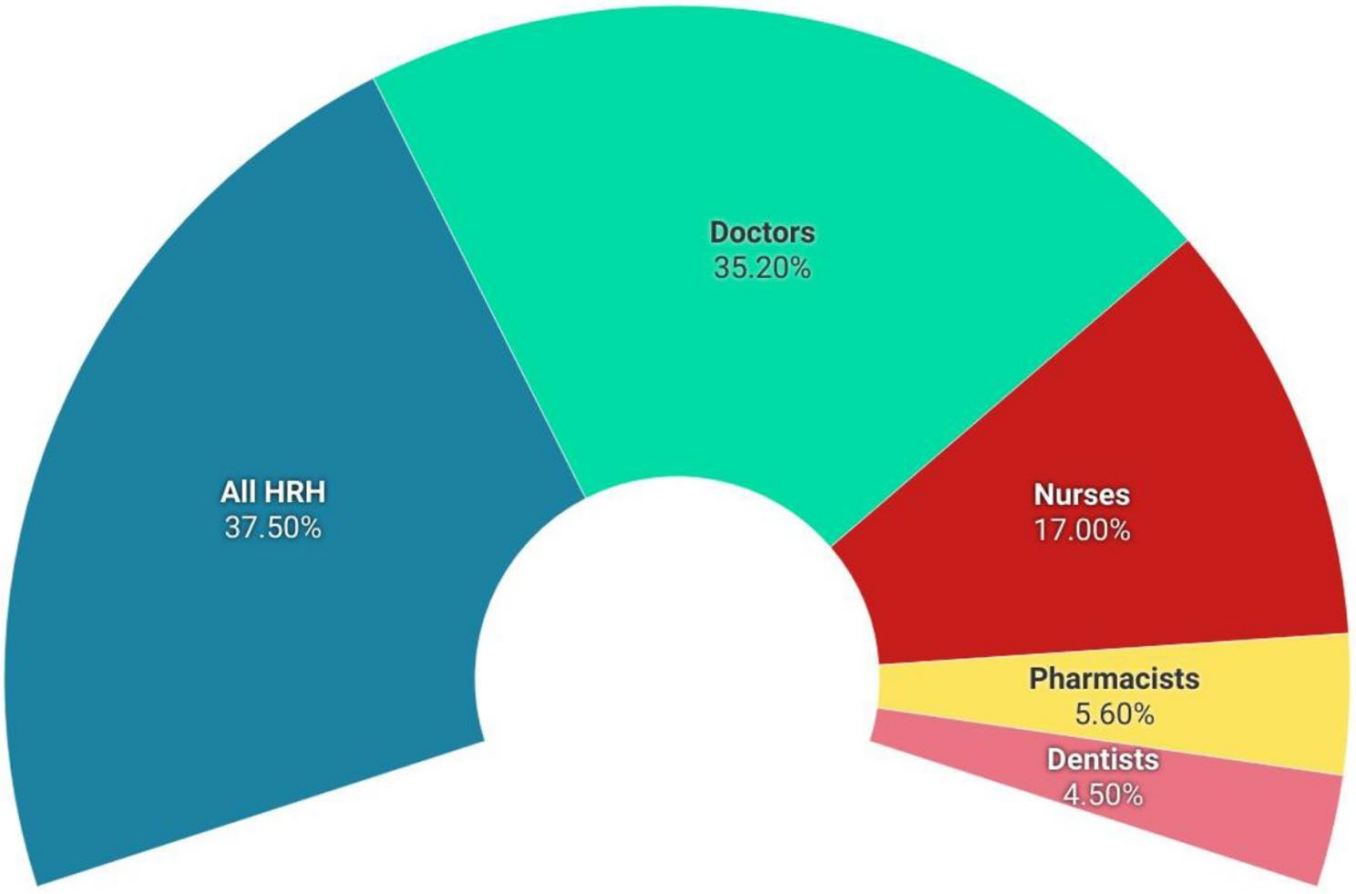
Distribution of studies according to HRH

Studies enumerating more than one cadre were categorized as all HRH (*n* = 33, 37.5%) in the present study. 35.2% (*n* = 31) studies were conducted on doctors, 17% (*n* = 15) on nurses, 5.6% (*n* = 5) on dentists and 4.5% (*n* = 4) on pharmacists (Fig. 3).

**Fig. 4:**
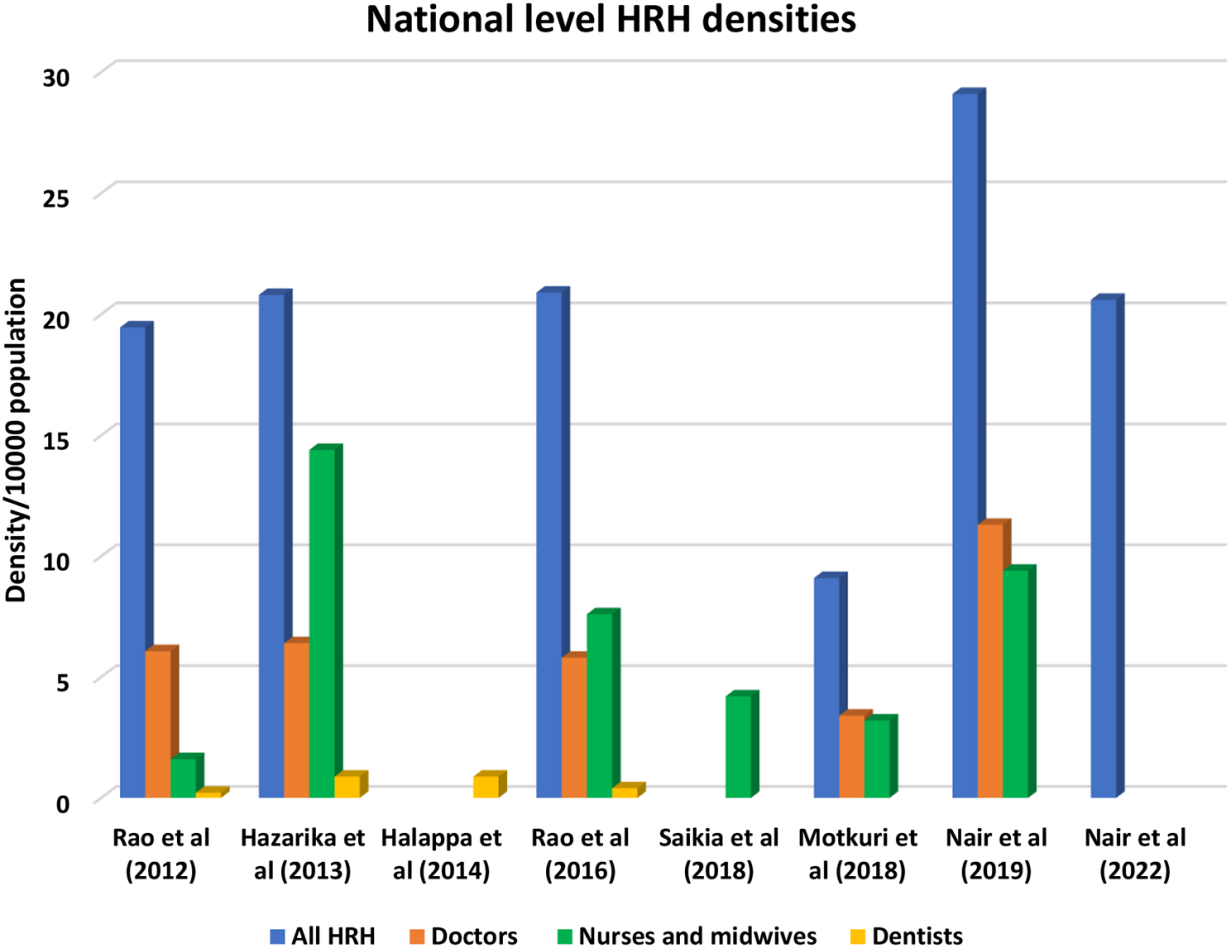
National Level HRH densities. NSSO = National Sample Survey Organization; MCI = Medical Council of India; INC = Indian Nursing Council; IMSPI = Indian Ministry of Statistics and Programme Implementation; WBO = World Bank Open Data; NHP = National Health Profile 2017; ABCE project surveys: Access, Bottlenecks, Costs, and Equity (ABCE) project surveys

Eight cross-sectional national-level studies reporting the HRH data in India are presented in this scoping review. The HRH concentrations are compiled in Fig. 3. Comparable figures have been derived from the data since the WHO views the doctor, nurse, and midwife cadres as vital HRH. The key data sources for the studies were considered to be estimates from the Census, the National Sample Survey Organisation (NSSO), professional registration bodies, Population data and health-professional statistics, the National Health Profile, and the Indian Ministry of Statistics and Programme Implementation’s 2011 Report on Health and Family Welfare. Results depict an increase in the density of all HRH and doctors from 19.46 to 6.07 in 2012 to 29.1 and 11.3 in 2019 respectively (Fig. 4).

### Thematic analysis

The thematic analysis of data regarding the second research question yielded five kinds of HRH-related problems that are causing a scarcity of HRH in India. The outcomes of each study are described in Supplementary Tables 3 and codes identified under each theme is shown in Supplementary Table 4 [10, 16–102]. A summary of these themes is provided in Table 2 below:

**Table 2:** Reasons for shortage

Theme (Reason for HRH shortage)	Examples	No. of studies	References
1. Inadequate HRH production	Due to an inadequate number of healthcare educational institutions in India, there is a lack of healthcare professionals	**10**	[65, 66, 74, 75, 80, 81, 88, 102]
	The current medical colleges are also underfunded and underequipped, which results in substandard training of healthcare professionals		
3. Job dissatisfaction	Poor physical work conditions, autonomy, unhealthy relations with colleagues, boss and management	**39**	[10, 16, 18, 19, 22, 24, 26–28, 30, 34, 39, 41, 43, 46, 51, 53, 54, 58, 59, 61–66, 71, 75, 78, 81, 84, 88, 89, 91, 92, 94, 96, 99, 102]
	Inadequate salary, no opportunities for promotion, no respect, and non-recognition leads to dissatisfaction		
	Ineffective management of HRH causes major stress reactions, which can further contribute to job discontent, burnout, and poor QOL		
	Healthcare professionals in India also face limited career growth opportunities, leading to a lack of motivation and a shortage of skilled healthcare professionals		
4. Brain Drain	Financial reasons, Social and professional ambitions, religious and gender-related issues, family support, and assistance from migrant networks are the key reasons for migration.	**10**	[17, 31, 37, 48, 68, 70, 80, 85, 90, 97]
	It is difficult for India to retain skilled medical personnel due to the movement of Indian HRH to nations with higher incomes, which affects government efforts to make healthcare more accessible throughout the nation.		
5. Regulatory concerns	Workforce expansions are not at pace with population growth and changing dynamics of regional disease burden.	**33**	[21, 23, 25, 29, 30, 32, 33, 35, 36, 38, 42–45, 47, 49, 50, 55–57, 67, 69, 73, 77, 79, 82, 83, 86, 95, 99, 100, 102]
	Incredibly complex, non-transparent, and dispersed recruitment rules, slow and extremely erratic recruitment process, health department’s protracted delivery of wage benefits and service regularisation, unequal opportunities with regard to job stability, no wage benefits, and non-acknowledgment of prior work experience are key regulatory issues		
	Regular vacancy planning is not done at the district level. The district health societies hire only contractual staff at the district level.		
	Regularly updated HR planning is not performed, and as a result, actual HR requirements are not calculated, creating a backlog of shortages.		
	Current central civil service rules, recruitment methods, appraisal systems, reward and punishment, and so on are insufficient to address human resource management issues.		
	Uneven distribution of doctors in health centers		
	Doctor distributional disparities are the most pronounced and have a substantial impact on health outcomes.		
6. Lack of training, monitoring and evaluation	Inadequate capacity for routine supervision There are no on-site mentors or technical assistance for health professionals with limited skills.	6	[34, 35, 40, 52, 60, 83]
7. Regulatory issues	Workforce expansions are not at pace with population growth and changing dynamics of regional disease burden.	15	[23, 25, 29, 30, 32, 42, 43, 45, 49, 55–57, 69, 77, 102]
	Incredibly complex, non-transparent and dispersed recruitment rules, slow and extremely erratic recruitment process, health department’s protracted delivery of wage benefits and service regularisation, unequal opportunities with regard to job stability, no wage benefits, and non-acknowledgement of prior work experience are key regulatory issues		
	Regular vacancy planning is not done at the district level. The district health societies hire only contractual staff at the district level.		
	Current central civil service rules, recruitment methods, appraisal systems, reward and punishment, and so on are insufficient to address human resource management issues.		

A detailed description of all the themes are given below:

- **Theme 1: Inadequate HRH Production and recruitment**

“Inadequate HRH production” emerged out to be the first theme in the present review. Eight studies reported this theme as one of the reasons for the HRH shortage in India. With nearly 1.3 billion citizens, India is the second-most populated nation in the world. This puts a tremendous amount of strain on the healthcare system, which needs a sizable number of healthcare staff to meet the population’s healthcare needs [80, 88]. Also, India suffers from serious health disparities, with a large divide between urban and rural areas as well as across various states [65, 66, 88]. Healthcare professionals are in insufficient supply in many rural areas and several states, and their distribution is not equitable for instance, in urban Madhya Pradesh (MP), there are 120 doctors per 100,000 people, whereas in rural MP, there are only 12 doctors per 100,000 people [22]. With an aging population, India is going through a demographic transformation as well [65, 66, 74, 75, 81]. The demand for healthcare services will rise as a result, especially for geriatric care, which calls for a qualified staff [81, 88]. With a large number of people coming from other nations for medical treatment, India has become a well-liked location for medical tourism. The need for healthcare personnel has expanded as a result, especially in specialized professions. However, there is a limited number of postgraduate (PG) seats in medical courses which makes it challenging to maintain supply as per the demand [88]. Moreover, there is a lack of a centralized HRH database which hinders effective planning and HRH deployment in certain locations [88].

- **Theme 2: Job dissatisfaction**

Thirty-nine studies reported that job dissatisfaction is a major contributor to India’s shortage of Human Resources for Health (HRH). In India, a large number of healthcare professionals operate in subpar facilities with insufficient equipment. Burnout, stress, and work unhappiness may result from this [10, 16, 24, 26, 39]. Further, healthcare professionals are frequently underpaid, especially in the public sector. Many Indian healthcare employees believe that there are few opportunities for professional growth, which might cause them to feel unmotivated and dissatisfied with their jobs [63, 71, 75]. Therefore, in order to address the lack of HRH in India, it is imperative to address the issue of work unhappiness among healthcare professionals.

- **Theme 3: Brain Drain**

Ten studies reported that for emerging nations like India, where the loss of trained individuals can have a large influence on economic growth and development, brain drain can be a serious issue [17, 31, 37, 48, 68, 70, 80, 85, 90, 97]. The term “brain drain” describes the emigration of highly educated and competent people from one nation to another [31, 37]. The desire for better employment possibilities is one of the primary causes of brain drain. Many highly qualified individuals leave their home nation in quest of better-paying work and living conditions [68, 70]. Another factor in brain drain is a lack of employment prospects in a particular field or business. Skilled workers may search for chances abroad if they are unable to obtain employment in their field at home.

In some situations, the pursuit of educational possibilities can result in brain emigration. Professionals with advanced degrees may travel abroad to complete their studies or receive training in an area that is not offered or accessible in their native country [68, 70, 80, 85, 90, 97].

- **Theme 4: Regulatory concerns**

“Regulatory concerns” emerged to be another important theme. Thirty-three studies reported that to ensure an adequate supply of HRH, regulatory concerns must be resolved. A lack of qualified healthcare personnel is caused by inadequate staffing and training regulations. The health department’s protracted delivery of wage benefits and service regularisation, unequal opportunities with regard to job stability, no wage benefits, and non-acknowledgment of prior work experience, extremely complex and dispersed recruitment rules, a slow and erratic recruitment process are the key regulatory issues indicated by majority of studies [36, 42, 43, 45, 47, 49, 50, 67, 69].

- **Theme 5: lack of training, monitoring and evaluation**

Six studies stated that in the absence of proper training healthcare professionals might not be able to pick up the skills and information required to do their professions well. This may result in a lack of qualified healthcare professionals who can deliver high-quality care. Further, without efficient monitoring and evaluation methods, it may be challenging to pinpoint the areas in which healthcare staff need more assistance or training [34, 35, 40, 52, 60, 83]. The expertise and abilities of healthcare professionals may not match the population’s demands as a result, which could contribute to the shortage of HRH.

- **Theme 6: Regulatory issues**

15 studies Workforce expansions are not at pace with population growth and changing dynamics of regional disease burden. Incredibly complex, non-transparent and dispersed recruitment rules, slow and extremely erratic recruitment process, health department’s protracted delivery of wage benefits and service regularisation, unequal opportunities with regard to job stability, no wage benefits, and non-acknowledgement of prior work experience are key regulatory issues. Regular vacancy planning is not done at the district level. The district health societies hire only contractual staff at the district level. Current central civil service rules, recruitment methods, appraisal systems, reward and punishment, and so on are insufficient to address human resource management issues.

## Discussion

This scoping review is aimed at offering a thorough comparative evaluation of research conducted on the scarcity of human resources for the health sector in India, and an analysis of the deficit distribution throughout cadres. Numerous aspects of HRH in India, as well as current and upcoming issues that must be resolved to enhance the availability of health personnel, both nationally and at the state level, have been highlighted. A total of 88 studies that reported HRH densities and reasons for HRH shortages indicated variations in their data sources like sample surveys, censuses, and registries. Thirty-three national level studies examining the HRH data in India were identified. Findings reveal that the number of doctors, nurses, and midwives in India is only one-quarter of the World Health Organization guideline of 2.3/1000 people, indicating a severe general deficit of health professionals. The workforce has an inefficient skill mix because there are at least as many doctors as nurses. Just one-third of the work force are women. Most workers reside in cities and are employed by the private sector [37].

Studies by Singh et al. and Rao et al. also report overall low numbers of qualified health workers, a high presence of unqualified health workers, particularly in rural areas, and significant differences in qualified health worker distribution between urban and rural areas [52, 69]. A framework has been developed to identify the reasons for the underlying shortage, in the form of six themes along with proposed strategies and actions that can assist governments, policy makers and health agencies in planning, creating, and executing efficient strategies for achieving a sustainable health workforce and UHC. It is clear that there are shortages of health workers in some regions of India and in some speciality fields, but it is challenging to assess the scope and type of such shortages due to a dearth of research and health statistics. There is a glaring lack of clarity on whether a connection exists between these shortages and global migration. Although there is no specific policy agenda to control health worker migration in general, policy responses to migration of health workers are typically integrated into wider processes aimed at managing the health workforce. India’s decision-makers have divergent opinions on whether it is necessary or desirable to restrict immigration [78].

India’s health care systems and services are still developing, therefore facing issues like lack of skilled workers, absenteeism, inadequate infrastructure, and care quality [66, 80, 99]. One important determinant of the availability of health workforce is the density of the health personnel, relative to the population. Poorer health and service utilisation results are found in states with lower health worker densities [32]. The findings also revealed that public hospital employees were more satisfied with their recruitment and selection process, less committed to their organisation, and had lower levels of occupational stress than private hospital employees [41, 49]. Enhancing working conditions, providing the bare necessities in terms of supplies and equipment, providing possibilities for professional growth, and strengthening supervision may prove to be equally significant in boosting employee retention in a desperate human resources situation. Furthermore, there is an unequivocal need to improve the quality of the output in terms of an explicitly stated and standardized competency framework tailored to the Indian context.

Forty-seven studies focussing on the HRH of a single state were conducted at the state level. Studies conducted in Gujarat reported that incredibly complex, non-transparent and dispersed recruitment rules, slow and extremely erratic recruitment process, the health department’s protracted delivery of wage benefits and service regularisation, unequal opportunities with regard to job stability, no wage benefits, and non-acknowledgement of prior work experience are the key factors influencing the HRH in most of the states. As opposed to extrinsic motivation, intrinsic motivation is more crucial. In order to meet the demands of service providers in terms of motivation, state health departments must address the motivation of health service providers by designing a set of strategies. State health departments, lawmakers, and reformers need to create management strategies that address both intrinsic and extrinsic motivational factors [77, 89].

Similarly, the study conducted in Mumbai reported that the high rate of burnout syndrome among resident doctors in public sector hospitals had a negative impact on the physical and mental health of medical professionals and lowered their motivation and productivity at work [81]. Migration of Indian HRH to nations with higher incomes emerged as another significant factor that impacts HRH retention in India. A study conducted at the international level, including India, indicated that it is difficult for India to retain skilled medical personnel due to the movement of Indian HRH to nations with higher incomes, which affects government efforts to make healthcare more accessible throughout the nation [17]. Low pay and unfavourable working conditions, particularly in the private sector, are the main drivers of Indian HRH leaving India for other countries [21, 75, 78, 85].

Another significant concern identified by a majority of the research is a lack of HRH databases [23, 28, 49, 82, 90]. There is a backlog of shortages because actual HR requirements are not assessed as a result of the lack of routinely updated HR planning. Although having a big workforce, the state health department lacks a specialised HR department to offer assistance with a variety of HR responsibilities. Ad hoc workers who put in long hours do not receive the same perks as regular workers. The existing sanctioning standards require an evidence-based update. Workload-based HRH deployment in different regions will guarantee sufficient availability and equitable distribution, which are required to raise the general standard of healthcare.

Inequitable distribution of HRH, lack of training, limited and poor supervision turned out to be another important factor that influence HRH in India. The most apparent doctor distributional gaps have a significant impact on health outcomes. Lack of or unequal distribution of the medical workforce may also result in inefficient utilisation of physical facilities and equipment, making the infrastructure and equipment investments useless. Zurn et al. also reported that inequitable distribution of healthcare manpower is an important challenge for health policymakers [103].

Health planners and managers must pinpoint crucial aspects, including training opportunities, which can be methodically handled at the management and policy level in order to solve this issue. Quantification, understanding, and accessibility of crucial elements can surely aid in the development of efficient administrative and human resource policies. The population is growing, and the dynamics of the regional illness burden are changing, yet workforce expansion is not keeping up. The health department’s lengthy delivery of wage benefits and service regularisation, unequal opportunities with regard to job stability, no wage benefits, and non-accountability of prior work experience are important regulatory issues. Further regulatory concerns include excessively complex, unclear, and dispersed hiring standards, a sluggish and inconsistent hiring procedure, and extremely slow and erratic recruitment rules [29, 45, 55, 56, 66]. Public-private partnerships (PPPs) are frequently employed to take advantage of the resources, skills, and knowledge of the private sector around the world [7]. In order to complement the public sector, the partnership may look into the resources and experience of the commercial sector. The findings of the present scoping review could assist decision-makers in deciding the future road map to accomplish the sustainable development goals. The study has a number of strengths. Firstly, it adds to the little body of knowledge on the shortage of HRH and the disparities in the publicly financed healthcare system in India. Secondly, the current study not only assessed the shortage of human resources for health (HRH) in India but also identified the key reasons for the shortage.

There are a few limitations as well. WHO’s methodology for determining HRH density requirement thresholds for doctors, nurses and midwives and dentists were the only considerations, thus leaving out other paramedical staff due to non-availability of data in most of the studies. The HRH system in India is divided into public and private sectors and, while the private sector lacks a formal hierarchy of structure, the public health system follows a three-tier model, with primary, secondary, and tertiary levels. In our review we witnessed a lack of literature depicting the shortages of public and private HRH. This is because we followed a scoping review approach and considered reasons for shortages published in the literature which may not be comprehensive. Another limitation is that, due to heterogeneity in the included studies, public and private sector and urban and rural comparisons couldn’t be estimated. Although we have tried to cover all the major databases, we might have missed out some of the important papers due to the non-responsiveness of authors in sharing the complete data. Furthermore, our scoping analysis did not explicitly analyse data from sources such as Rural Health Statistics, National Health Workforce Accounts, and the Periodic Labour Force Survey, which can give critical information on HRH. As a result, the assessment may not fully capture the detailed insights from these main data sources. Further research could benefit from a more direct examination of these primary data sources to gather a greater range of information about the health workforce.

The present scoping review has a few recommendations. Firstly, a comprehensive national database covering HRH cadres in public and private sectors could accurately track the state of HRH in India and make necessary policy changes to improve it. The current skill mix is dominated by doctors and consists of fewer nurses. At the national level, there needs to be a focus on both retaining nurses in the workforce and significantly boosting nursing supply. More focus will be required on the unique role of task shifting and its effects on patient care and well-being. Reduce the current human resource shortfalls in public sector organisations, especially at the primary levels, by making recruiting processes more effective through walk-in interviews or contractual/flexible norms of involvement. To strengthen the HRH in India, we require a comprehensive strategy that covers finance, infrastructure, working conditions, gender and social inequities.

## Conclusion

This scoping review reveals that there has been a persistent shortage and inequitable distribution of human resources in India over the years, with the rural expert cadres experiencing the most shortage. The critical challenges in India’s Human Resources for Health (HRH), highlight inadequate HRH production and recruitment, job dissatisfaction, brain drain, regulatory issues, and training deficits as key factors contributing to the HRH shortage. To address these multifaceted challenges, the health department must establish a productive recruitment system to achieve long-term solutions Having clear guidelines for managing human resources and being transparent in how these are put into practice would enhance governance and foster trust among healthcare professionals, thus motivating them to work in the public sector. Therefore, the optimal management of these challenges has the power to promote retention by boosting motivation and preventing voluntary turnover.

### Electronic supplementary material

Below is the link to the electronic supplementary material.

Supplementary Material 1

## Data Availability

All data are included in the manuscript. Remaining data can be provided on reasonable request by corresponding author.
