# Supplementary material for: Human resource shortage in India’s health sector: a scoping review of the current landscape
Source: BMC Public Health. 2024 May 21;24:1368. doi: 10.1186/s12889-024-18850-x (PMC11110446; doi:10.1186/s12889-024-18850-x)
Supplement: Supplementary file 1 — Supplementary Material 1 [file 12889_2024_18850_MOESM1_ESM.docx]

**Supplementary Table 1 A: Search Strategy for Cochrane**

| **Domains** | **Keywords** |
| --- | --- |
| Human resources for health | (Human resources) OR (HRH) OR (health system) OR (Healthcare Planning) OR (health services) OR (Primary health care system) OR (Universal health coverage) |
| Health workforce | (Health workers) OR (Multi-purpose health workers) OR (Village Health Guides) OR (Health personnel) OR (Community health workers) OR (health associated workers) OR (Accredited Social Health Activist) OR (health care workers) OR (Health service providers) OR (Health worker shortage) OR (Community Health Workers) OR ("Health Personnel") OR ("Health Workforce") OR (Health Services) |
| Doctors | (Dentists) OR (Nurses) OR (Midwives) OR (Traditional & faith healers) OR (AYUSH) OR (Allopathic) OR (Dental auxiliaries) OR (Specialists) OR (Physicians) OR (Ayurvedic) OR (Homeopathy) |
| Shortage | shortage OR rural deployment OR medically underserved areas |
| Health | Public Health |
| India | India OR (Rural India) OR (South-East Asia) |

**Supplementary Table 1 B: Search Strategy for Embase**

| **Domains** | **EMBASE Keywords** |
| --- | --- |
| Human resources for health | (HRH) OR (health system) OR (resources of health) OR (health services) OR (Primary health care system) OR (Universal health coverage) OR (Universal Health Insurance) |
| Health workforce | 'health care personnel' OR 'health workforce' OR 'health auxiliary' |
| Doctors | (Dentists) OR (Nurses) OR (Midwives) OR (Traditional & faith healers) OR (AYUSH) OR (Allopathic) OR (Dental auxiliaries) OR (Specialists) OR (Physicians) OR (Ayurvedic) OR (Homeopathy) |
| Shortage | shortage OR rural deployment OR medically underserved areas OR 'job satisfaction' OR recruitment |
| Health | 'public health' OR 'public health service' |
| India | India OR (Rural India) OR (South-East Asia) |

**Supplementary Table 1 C: Search Strategy for Web of Science**

| **Domains** | **Keywords** |
| --- | --- |
| Human resources for health | TS=((HRH) OR (health system) OR (resources of health) OR (health services) OR (Primary health care system) OR (Universal health coverage) OR (Universal Health Insurance)) |
| Health workforce | TS=((Health workers) OR (Multi-purpose health workers) OR (Village Health Guides) OR (Health personnel) OR (Community health workers) OR (health associated workers) OR (Accredited Social Health Activist) OR (health care workers) OR (Health service providers) OR (Health worker shortage) OR (Community Health Workers) OR (Health Personnel) OR (Health Workforce) OR (Health Services)) |
| Doctors | TS=((Dentists) OR (Nurses) OR (Midwives) OR (Traditional & faith healers) OR (AYUSH) OR (Allopathic) OR (Dental auxiliaries) OR (Specialists) OR (Physicians) OR (Ayurvedic) OR (Homeopathy)) |
| Shortage | TS=(shortage OR rural deployment OR medically underserved areas) |
| Health | TS=(Public Health) |
| India | TS=(India OR (Rural India) OR (South-East Asia)) |

**Supplementary Table 1 D: Search Strategy for Scopus**

| **Domains** | **Keywords** |
| --- | --- |
| Human resources for health | (Human resources) OR (HRH) OR (health system) OR (Healthcare Planning) OR (health services) OR (Primary health care system) OR (Universal health coverage) |
| Health workforce | (Health workers) OR (Multi-purpose health workers) OR (Village Health Guides) OR (Health personnel) OR (Community health workers) OR (health associated workers) OR (Accredited Social Health Activist) OR (health care workers) OR (Health service providers) OR (Health worker shortage) OR (Community Health Workers) OR ("Health Personnel") OR ("Health Workforce") OR (Health Services) |
| Doctors | (Dentists) OR (Nurses) OR (Midwives) OR (Traditional & faith healers) OR (AYUSH) OR (Allopathic) OR (Dental auxiliaries) OR (Specialists) OR (Physicians) OR (Ayurvedic) OR (Homeopathy) |
| Shortage | shortage OR rural deployment OR medically underserved areas |
| Health | Public Health |
| India | India OR (Rural India) OR (South-East Asia) |

**Supplementary Table 1 E: Search Strategy for all databases**

| **Databases** | **No. of hits** | | | | **Total hits** |
| --- | --- | --- | --- | --- | --- |
|  | **1 AND 4 AND 6** | **2 AND 4 AND 6** | **3 AND 4 AND 6** | **4 AND 5 AND 6** |  |
| PubMed | 557 | 558 | 264 | 565 | 1944 |
| Cochrane | 113 | 106 | 83 | 115 | 417 |
| EMBASE | 371 | 107 | 248 | 364 | 1090 |
| WoS | 761 | 591 | 290 | 425 | 2067 |
| Cinahl | 256 | 102 | 238 | 105 | 701 |
| Scopus | 407 | 388 | 253 | 339 | 1387 |
| EBSCO Global health + Global health Archives | 857 | 194 | 335 | 491 | 1877 |
| Additional Sources |  |  |  |  | 97 |
| **Total** |  |  |  |  | **9580** |

**Supplementary Table 2: Study Characteristics**

| **Author and Year** | **Location (State/district)** | **Study setting** | **Type of data** | **Study design** | **Sample size** | **Data collection tool** | **Data Analysis technique** | **Cadres** | **Index/Guidelines** | **Career Stages** | **Employment status** |
| --- | --- | --- | --- | --- | --- | --- | --- | --- | --- | --- | --- |
| Zeighami et al., 1978 [16] | India, Pakistan Philippines | Survey | Primary data | Cross sectional survey | 84 | Questionnaire | Descriptive statistics | Physicians | NA | Exploratory To Decline Level | Employed |
| Goyal et al., 1979 [17] | Haryana | Health centres of department of health services Haryana. | Secondary data | Secondary research | 9 health centres of department of health services Haryana. | NA | The heuristic method | Doctors | MCI guidelines | Not Mentioned | Employed |
| Astor et al., 2005 [18] | Multicentric study conducted in India, Pakistan, Colombia, Philippines, and Nigeria | University/Medical schools | Primary data | Cross sectional survey | 644 physicians | Questionnaire | Chi-square test | Physicians | MCI guidelines | Not Mentioned | Employed |
| Bhat et al., 2005 [19] | Chhattisgarh | CHC’s | Primary data | Mixed method study (both qualitative and quantitative) | Qualitative: 10 quantitative: 70 | Focused group discussions | Student t test, Regression analysis | District and State level health officials, doctors of CHC | NR | Mid-Career To Late Career | Employed |
| Mullan, 2005 [20] | International level study including India | Four countries including USA, UK, Canada and Australia | Secondary data | Secondary research | NA | American Medical Association for America, The National Health Service of UK, Southam Medical Database and 2002 Canadian Post-MD Education Registry and Labour Force and Rural Health Unit of the Australian Institute of Health and Welfare (AIHW) for Australia | Descriptive statistics | Physicians | NR | NM | NA |
| Thomas, 2006 [21] | Delhi | Four government-owned hospitals, eight private hospitals, four schools of nursing and four colleges of nursing located in various parts of Delhi | Primary data | Cross sectional survey | 448 | Questionnaire | Descriptive statistics, chi square test | Nurses | NA | NM | Employed |
| De Costa et al., 2007 [22] | Madhya Pradesh | Village in rural areas ward in urban areas | Primary data | Cross-sectional survey | 24,807 doctors, 55393 traditional birth attendants and 89090 unqualified providers | Field survey | Descriptive statistics | Physicians in urban areas and qualified paramedical staff | MCI and INC guidelines | Not Mentioned | Employed |
| Katrak, 2008 [23] | Madhya Pradesh | Ujjain district in Madhya Pradesh | Secondary data | Secondary research | NA | India health information (2002) | Simulation exercises | Physicians, nurses, and midwives | NR | NM | NA |
| Alonso et al., 2009 [24] | International level study including India | National Health Service acute trust in London | Primary data | Both longitudinal and cross sectional study | 6 internationally recruited nurses from India and 15 from Philippines | Face-to-face longitudinal and cross-sectional interviews | Intra-case and cross-case comparisons | Nurses | INC guidelines | Not Mentioned | Employed |
| Bhandari et al., 2010 [25] | Delhi | Four CGHS zones of Delhi | Primary data | Cross sectional survey | 250 healthcare providers from 20 dispensaries | Interviews and standardised tool developed by Peter Warr, John Cook and Toby Wall | Descriptive statistics | Doctors, nurses and paramedics | NR | Mid-Career To Late Career | Employed |
| Bhattacharya et al., 2011 [26] | National level study (Northern India) | Public as well as private hospitals covering rural and urban regions | Primary data | Cross-sectional survey | 807 | Structured questionnaires | Factor analyses, Chi-square tests | Doctors, nurses, paramedics, and administrators | MCI and INC guidelines | Exploration To Establishment | Employed |
| Sodani et al., 2011 [27] | Bharatpur, Rajasthan | CHC’s | Primary data | Cross sectional survey | 13 CHC’s | Observation of records/register at CHC's | Descriptive statistics (frequency distribution, tables) | Specialist at CHC(General surgeon, physician, paediatrician, OBG, MO) Support staff (Nursing staff, lab technician, radiographer, pharmacist) | Revised Draft of IPHS for Community Health Centers 2010 | NM | Employed |
| Aggarwal et al., 2012 [28] | Punjab and Haryana | Four private dental institutions | Primary data | Cross sectional survey | 400 dental students | Structured questionnaire | Chi-square test | Dental students | Dental Council of India | Exploration | Unemployed |
| Hagopian et al., 2012 [29] | Orrisa | India Census (Government of India 2001), routine health information data from health units in Ganjam District, the Child Survival and Safe Motherhood register of health workers, the district’s health ‘program implementation plan’, and a 2005–07 yearbook of ‘special information on health infrastructure of Orissa’. Primary data from 6 community health centres, six primary-care level health centres , and six sub health centres | Primary as well as Secondary data | Mixed method study (both qualitative and quantitative) | 24 interviews with physicians, staff nurses, lady health visitors and laboratory technicians, 30 interviews with auxiliary nurse midwives, 10 interviews with mothers. | Interviews and focus group discussions | Calculation methods recommended in the WHO’s WISN guide | Doctors and nurses | MCI and INC guidelines | NR | Employed |
| Kadam et al., 2012 [30] | Odisha | Sub centres, primary health centres and community health centres in 6 districts of Odisha | Primary as well as Secondary data | Mixed method study (both qualitative and quantitative) | 226 doctors, nurses, pharmacists, multipurpose health workers (MPHW) and laboratory technicians. | Semi-structured interviews | Descriptive statistics | Doctors, nurses, pharmacists, multipurpose health workers (MPHW) and laboratory technicians. | NR | NM | Employed |
| Murthy et al., 2012 [31] | National level study | Andhra Pradesh and Uttarakhand. | Primary data | Cross sectional survey | 68 (23 graduates doing medical internship, 19 postgraduate medical students and 26 in-service doctors) | Semi-structured interviews | Thematic analysis | Doctors | NR | Exploration To Mid-Career | Both employed and students |
| Rao, 2012 [32] | Uttarakhand and AP | PHC | Primary data | Qualitative study | Medical Students-308, in-service health workers-460 (doctors and nurses) | Field survey using questionnaires | Bivariate probit and mixed logit regression | Medical students, in-service health workers(doctors and nurses) | NA | Exploration | Unemployed |
| Rathore et al., 2012 [33] | Udaipur Rajasthan | Maharana Bhopal Government Hospital a | Primary data | Cross sectional survey | 60 nurses | Standard Shift work Index manual | Descriptive statistics (frequency distribution, tables, and graphs) | Nurses | NR | Establishment To Decline | Employed |
| Saha et al., 2012 [34] | Dahod Gujrat | Govt and private sectors | Primary data | Survey | 36 medical graduates from government sector and 19 medical graduates working in the private sector | Questionnaire | Descriptive statistics, F test | Class 1 specialists, allopathic physicians, BAMS, BHMS | NR | NM | Employed |
| Saini et al., 2012 [35] | Delhi, Rohtak | Medical colleges | Primary data | Cross sectional survey | 201 UG students | Pre-tested semi-open-ended questionnaire | Descriptive statistics(percentage), chi square test | Medical students | NR | Exploration | Unemployed |
| Tiwari et al., 2012 [36] | Rajasthan | District Hospital, Sub-Divisional Hospital, Community Health Centres and Primary Health Centres | Primary data | Cross sectional survey | 40 | Interview | Descriptive statistics | Specialists and medical officers | NR | NM | Employed |
| Rao et al., 2012 [37] | National level study | NA | Secondary data | Secondary research | NA | 2001 Census of India and the 61st round (July 2004-June 2005) of the National Sample Survey (NSS) on ‘Employment and Unemployment’ | Descriptive statistics | Health workers included allopathic physicians, AYUSH practitioners, nurses and midwives, dentists, pharmacists, others (including the paramedical support staff ) and other practitioners of traditional medicine | The National Occupational Classification (NOC) codes were | NA | NA |
| Abraham et al. , 2013 [10] | Mangalore | Five colleges and two hospitals | Primary data | Cross sectional survey | 50 nurse educators and 50 staff nurses | Demographic proforma, job satisfaction inventory, Shirom Melamed Burnout Inventory and WHOQOL-BREF | Frequency, percentage tables, Independent ‘t’ test, Chi-square test and Karl Pearson coefficient of correlation. | Nursing | INC guidelines | Exploration To Mid-Level | Employed |
| Hazarika, 2013 [38] | National level study | Data from Indian Ministry of Statistics and Programme Implementation's 2011 Report on Health and Family Welfare. | Secondary data | Secondary research | NA | Secondary data | Lorenz curves and Gini indices | Entire health workforce | MCI, DCI, and INC guidelines | NA | Employed |
| Ramesh et al., 2013 [39] | Bangalore | Medical college hospital | Primary data | Cross‑sectional survey | 671 nurses | The modified version of QWL questionnaire | Independent ‘t’ test and correlation analysis | Nurses | NR | Exploration To Mid-Career | Employed |
| Pandey et al., 2013 [40] | Madhya Pradesh | Hospital-based study | Both Primary and secondary data | Cross‑sectional survey | Six laboratory technicians | Questionnaire | Rate ratio and proportions, | Lab Technician, WISN, secondary sources/record review of annual hospital statistics. | NM | NM | NM |
| Kumar et al., 2013 [41] | Delhi | Primary Urban Health Centres and dispensaries under the Directorate of Health Services | Primary data | Descriptive study | 333 ,Medical Officers (101), ANM’s (114), Pharmacists (85), Lab Assistants and Lab Technicians (33) | Semi structured interview schedule | Descriptive analysis, tables | Medical Officers , ANMS, Pharmacists, Lab Assistants and Lab Technicians | NA | NA | Employed |
| Ramani et al., 2013 [42] | Uttarakhand, AP | Ayurvedic, Allopathic, Nursing Medicine Schools and PHC’s | Secondary data | Qualitative study | 88 students, allopathic, ayurvedic doctors and nurses | In depth interviews | Generic thematic analysis technique | Students, allopathic, ayurvedic doctors and nurses | NA | NA | Employed |
| Rao et al., 2013 [43] | Uttarakhand and AP | PHC, sub istrict hospitals | Secondary data | Qualitative study | 80 | Questionnaire | Descriptive analysis, tables, logistic regression analysis | Medical and nursing students, doctors, and nurses | NA | Exploration To Mid-Career | Employed |
| Sodani et al., 2013 [44] | Bharatpur, Rajasthan | CHC, PHC's | Primary data | Cross sectional survey | 89 health providers including 21 medical officers and 68 nursing staff | Interview | Descriptive statistics (frequency distribution, tables) | Human resources at CHC(Gen surgeon, physician, OBG, paediatrics, anaesthetist, MO, nursing staff, pharmacist, lab technician, radiographer) Human resources at PHC (MO, staff nurse, pharmacist, lab technician) | Indian Public Health Standards (IPHS) 2010 | NM | Employed |
| Purohit et al., 2014 [45] | Multicentric study conducted in Gujarat, Madhya Pradesh, and Orissa. | Primary Health Centres, Community Health Centres and District Hospitals | Primary data | Cross sectional survey | 92 in-service government Mos (52 from Gujarat, 22 from MP and 18 from Orissa). | Questionnaire by Pareek | Discrete choice models, Kendall’s coefficient of concordance | Medical officers | MCI guidelines | Not Mentioned | Employed |
| Garner et al., 2014 [46] | National level study | A hospital-based, private mission non-profit school of nursing in Bengaluru | Primary data | Qualitative participatory action research (Photovoice) | 14 undergraduate nursing students | Fujifilm Quick-Snap disposable camera to take photographs | Thematic analysis | Nurses | INC guidelines | Exploration | Employed |
| Gupta et al., 2014 [47] | Andhra Pradesh | Government and private hospitals | Primary data | Cross-sectional survey | Two hundred nurses | Structured questionnaire | Chi tests | Nurses | INC guidelines | NM | Employed |
| Halappa et al., 2014 [48] | National level study | NA | Secondary data | Secondary research | NA | Dental surgeons data from Indian Ministry of Statistics and Programme Implementation’s 2011 report, data on the number of dental educational institutions and number of admitted students from the DCI, Data regarding Public health dentists from the Indian Association of Public Health Dentistry information centre. | Situational analysis | Dentists | DCI guidelines | NA | Employed |
| Kumar et al., 2014 [49] | Delhi | Primary urban health centres | Secondary data | Descriptive observational study |  | Documents from DHS, CDMO, Director Family Welfare, Delhi State Health Mission, and website of Delhi Government Health Department were observed | Descriptive statistics | NA | NA | NA | NA |
| Bhatia et al., 2014 [50] | Gujarat | MOs from Primary Health Centres (PHCs) and Community Health Centres (CHCs) and AYUSH2 (Ayurveda, Yoga and Naturopathy, Unani, Siddha and Homoeopathy) doctors working with PHCs from one district in Gujarat | Primary data | Cross sectional survey | 29 regular MOs, 22 MOs on Ad hoc basis and five bonded doctors | A tool on motivation which is based on Herzberg’s theory of motivation developed by Pareek (2006). | Descriptive statistics, frequency distribution | MO's, AYUSH doctors | NM | NM | Employed |
| Sodani et al., 2014 [51] | Bharatpur, Rajasthan | PHC’s | Primary data | Case study | 19 PHC's | Observation of records/register at CHC's | Descriptive statistics (frequency table) | Human resources(medical officer, staff nurse, pharmacist, laboratory technician) | A facility assessment tool was developed referring the revised draft of IPHS for PHC's | NM | Employed |
| Singh et al., 2014 [52] | National level study | NA | Secondary data | Secondary research | NA | Data from the third wave of District Level Household and Facility Survey, the largest ever nation-wide survey of households and health facilities | Descriptive statistics, linear, logistic regression | Health workers | IPHA | NA | NA |
| Ahmad et al., 2015 [53] | Karnataka | Private and government pharmacy schools in Karnataka | Primary data | Cross sectional survey | 214 Pharmacy academicians | Questionnaire | Mann–Whitney U test and Kruskal Wallis tests | Pharmacists | Pharmacy Council of India | Exploration To Late Career | Employed |
| Amin et al., 2015 [54] | Gujarat | 9 NICUs across 6 cities of Gujarat | Primary data | Multicentre, cross sectional study | 129 nurses | Perceived Stress Scale and Professional Quality of Life Scale | Correlation coefficient and multiple regression | Nurses | INC guidelines | Exploration To Mid-Career | Employed |
| Bhattacharya et al., 2015 [55] | National Level study (urban locations of northern and central parts of India). | 20 Hospitals, multi-speciality nursing homes with a minimum 50 beds and providing tertiary services | Primary data | Cross-sectional survey | 586 | Structured questionnaire | Chi-square and t-tests, Multi-group path analysis | Doctors, nurses, and administrators | MCI and INC guidelines | Exploration To Late Career | Employed |
| Garner et al, 2015 [56] | Bangalore | Christian faith-based nursing school | Primary data | Qualitative participatory action research (Photovoice) | 14 Christian student nurses | Fujifilm Quick-Snap disposable camera to take photographs | Thematic analysis | Nurses | INC guidelines | Exploration | Unemployed |
| Nallala et al., 2015 [57] | Odisha | Three government and three private medical colleges of Odisha | Primary data | Cross‑sectional survey | 390 medical students | Semi‑structured questionnaire | Chi‑square test with a univariate analysis to estimate odds ratio. A multivariate analysis for significant variables to identify the associated factors | MBBS students | NA | NA | Unemployed |
| Yashika Negi & Rajni Bagga, 2015 [58] | Delhi | Tertiary care hospitals in New Delhi | Primary data | Cross‑sectional survey | 200 nurses | Work profile tool and standardized Maslach Burnout Inventory (MBI) tool to measure burnout. | Descriptive statistics, ANOVA and Correlation analysis was applied to examine the differences and relationship among work profile variables and burnout respectively | Nurses | NA | Exploration To Late Career | Employed |
| Saxena et al., 2015 [59] | National level study | Public and private hospitals | Primary data | Survey | 597 doctors, nurses, administrators | Questionnaire | Descriptive statistics, correlation, and regression | Doctors, nurses, administrators | NA | Exploration To Decline Stage | Employed |
| Zodpey et al., 2015 [60] | National level study | NA | Primary data | Exploratory study | NA | Annual estimates of different categories of HRH | Descriptive statistics | Doctors, nurses, auxiliary nurse midwifes, pharmacists | NA | NM | Employed |
| Ahmad et al., 2016 [61] | National level study | Web-based study | Primary data | Cross sectional web-based survey | 377 Indian pharmacists | Questionnaire | Mann–Whitney U test and Kruskal Wallis tests | Pharmacists | Pharmacy Council of India | Exploration To Late Career | Employed |
| Purohit et al., 2016 [62] | Gujarat | Primary Health Centres Community Health Centres and District Hospitals | Primary data | Cross sectional survey | 154 | Questionnaire | Factor analysis | Medical officers & nurses | MCI and INC guidelines | Not Mentioned | Employed |
| Purohit et al., 2016 [63] | Gujarat | Government health department at rural health centres from three districts of Gujarat | Primary data | Qualitative study | 24 | Interviews | Content analysis | Government medical officers | MCI guidelines | Establishment To Late Career | Employed |
| Pandey et al., 2016 [64] | Uttarakhand | Rural blocks of Uttarakhand | Primary data | Cross-sectional survey | 200 Asha’s | Structured questionnaire | Factor analysis and Post-hoc analysis | ASHA workers | NM | Exploration To Mid-Career | Employed |
| Kadam et al., 2016 [65] | Odisha | 30 administrative districts of Odisha, | Primary as well as Secondary data | Mixed method study (both qualitative and quantitative) | Four senior administrators and 90 doctors | Document review, key informant interviews and in-depth interviews with doctors. | Thematic analysis for qualitative data and descriptive statistics for quantitative data | Doctors | NM | Mid-Career To Late Career | Employed |
| Kadam et al., 2016 [66] | Maharashtra | Four districts: Dhule, Sangli, Nagpur, and Mumbai of Maharashtra | Primary as well as Secondary data | Mixed method study (both qualitative and quantitative) | 26 key informant interviews with 13 regulatory officials, 4 district-level representatives of the druggists and Chemists Association, 3 Ministry of Health and public health officials, 3 pharmaceutical producers, and 3 civil society activists. | Annual reports and government documents on the websites of central and state-level drug regulatory agencies (CDSCO, Maharashtra FDA, etc) and of ministries and related departments | Thematic analysis for qualitative data using MAXQDA version 10 and descriptive statistics for quantitative data | Regulatory officials, district-level representatives of the Druggists and Chemists Association, Ministry of Health and public health officials, pharmaceutical producers, and civil society activists. | NM | NM | Employed |
| Purohit et al., 2016 [67] | Delhi | Rural health centres from gov health departments | Primary data | Exploratory study | 19 MO’s | Document reviews, interview with key informants and MO's | Descriptive analysis, tables, thematic analysis | Mo's | NA | NM | Employed |
| Ranga et al, 2016 [68] | Uttar Pradesh, Bihar | 2 districts in UP and 1 in Bihar | Primary data | Longitudinal quantitative and spatial surveys | 2827 | Structured questionnaire, interviews, survey using hand held Garmin eTrex Vista Global Positioning System (GPS), | Dot charts, tables, histograms | Private non degree practitioners (PNDP) | NA | NM | Employed |
| Rao et al., 2016 [69] | National level study | NA | Secondary data | Secondary research | NA | National Classification of Occupations and National Industrial Classification codes | Descriptive statistics (density point estimates) | Allopathic doctors, nurses and midwives, dentists, AYUSH practitioners, health associates (paramedics or allied health professionals) and traditional practitioners | NA | NA | NA |
| Ambadekar et al., 2017 [70] | Maharashtra | Department of public health in rural parts of District Yavatmal, Maharashtra | Primary data | Cross sectional survey | 940 (96 medical officers and 844 allied health professional) | Structured self-administered questionnaire | Chi-square test | Medical officers & allied health professionals | MCI guidelines for Medical Officers | Not Mentioned | Employed |
| George et al., 2017 [71] | Delhi | Sarita Vihar subdivision of the south-east district | Primary data | Qualitative research | 19 clusters under Lal Kuan and 20 clusters in Sangam Vihar. | Focus-group discussions and interviews | NA | Community health workers | NA | Exploration To Mid-Career | Employed |
| George et al., 2017 [72] | International level study including India | NA | Secondary data | Secondary research | 5 countries | Data from pay commissions of 5 countries | Purchasing Power Parity (PPP) index | Nurses, doctors, and specialists including radiographer, anaesthetist, and general surgeon. | MCI and INC guidelines | Exploration | Employed |
| Janakiram et al., 2017 [73] | National level study | Public health dentists registered in Indian Association of Public Health Dentistry (IAPHD) working within India | Primary data | Cross-sectional survey | 580 dentists | Structured questionnaire | Descriptive statistics | Dentists | DCI guidelines | NM | Employed |
| Pandey et al., 2017 [74] | National level study | NA | Secondary data | Secondary research | NA | IPHS standards | Descriptive statistics | Paediatricians, obstetricians, and physicians | NA | NA | NA |
| Patil et al., 2017 [75] | Karnataka | Sub-centres of Belagavi District | Primary data | Cross‑sectional survey | NA | Structured questionnaire | Descriptive statistics | All specialists | NA | NA | NA |
| Pudpong et al., 2017 [76] | Bangladesh, China, India, Thailand, and Vietnam. | Rural, semiurban, and urban areas of India | Primary data | A descriptive comparative cross-sectional survey | 10,169 final-year nursing students (2880 from India) | Self-structured questionnaire | Descriptive analysis, tables, chi square test, multivariate analysis | Nurses | NA | Exploratory | Unemployed |
| Rajbangshi et al., 2017 [77] | Meghalaya and Nagaland | PHC, CHC, District hospitals | Secondary data | Qualitative study | 71 | Semi structured interview | Descriptive statistics, tables | Doctors, nurses, specialists | IPHS | Exploration To Mid-Career | Employed |
| Walton-Roberts et al., 2017 [78] | Kerala, Punjab, and Delhi | NM | Primary data | Cross sectional survey | 74 key stake-holders | Structured interviews | Descriptive statistics regression models, thematic analysis | Specialist physicians, nurses, midwives, dentists, pharmacists, dieticians, and other allied health therapies | NA | NM | Employed |
| Motkuri et al., 2017 [79] | National level study | NM | Secondary data | Secondary research | NA | NM | Descriptive statistics, tables, graphs | Doctors | NM | NA | Employed |
| Bates et al., 2018 [80] | International level study including India | FIP Collaborating Centre, UCL School of Pharmacy | Secondary data | Secondary research | Pharmacists Data collected in 2006, 2009 and 2012t | Data collected in 2006, 2009 and 2012 held on file at the FIP Collaborating Centre, University College London, School of Pharmacy and the FIP Education directorate | Descriptive statistics | Pharmacists | PCI guidelines | NA | NA |
| Saikia, 2018 [81] | National level study | Rural public health sector | Secondary data | Secondary research | NA | Data from Global Health Workforce Statistics published by the WHO, the World Bank Open Data and the National Health Profile 2017, Bulletin on Rural Health Statistics 2006 and 2016-17, Population Projections for India and States 2001–2026 | Number of nurses and midwives per 10,000 people, number of people served by one nurse or midwife and the ratio of nurses to doctors. Correlation analysis to examine the relationship between the density of nurses and health outcomes. | Nurses | INC guidelines | NA | Employed |
| Fundytus et al., 2018 [82] | International level study including LMICs, upper-middle–income countries (UMICs), and HICs. | 54 countries and two regions Caribbean and Africa | Primary data | Web based survey | 1,115 oncology physicians | Online electronic survey questionnaire | Chi tests to test for the difference in proportions, the Kruskal-Wallis test to compare ordinal and continuous data by income stratification. | Physicians | MCI guidelines | Mid-Career To Late Career | NM |
| Mathew, 2018 [83] | International level study including India | NA | Secondary data | Secondary research | NA | GLOBOCAN 2012 database | Descriptive statistics | Oncologists | NM | NM | NA |
| Oda et al., 2018 [84] | Tamil Nadu | Nursing Schools alumni | Primary data | Cross‑sectional survey | 265 nurses | Structured interviews | Descriptive statistics and probit analysis | Nurses | NM | Exploration To Mid-Career | Employed |
| Osman et al., 2018 [85] | International level study | Part of the Global Kidney Health Atlas project, under the umbrella of the International Society of Nephrology | Primary data | Cross‑sectional survey | 125 United Nations member states | Questionnaire | Descriptive statistics | Nephrologists and nephrology trainees | NM | Exploration To Late Career | NM |
| Tiwari et al., 2018 [86] | India | Medical Colleges and Institutions training HMPs in India in health (hospital) management/administration | Primary data | Cross sectional survey | NA | Service target approach and benchmark analysis | Descriptive statistics | Health management professional | NA | NM | Unemployed |
| Motkuri et al., 2018 [87] | 21 major states in India | NA | Secondary data | Secondary research | NA | Information sourced from Census of India | Descriptive statistics, tables, regression analysis | Health work force | NA | NA | NA |
| Singh, 2019 [88] | Uttar Pradesh | NA | Secondary data | Secondary research | 257 CHCs from DLHS-2, 693 CHCs from DLHS-3, and 920 CHCs from DLHS-4. | District-Level Household and Facility Survey rounds (2002–04, 2007–08, and 2012–13) | The Indian Public Health Standards for CHCs to measure shortages, Gini and Theil indices to measure inequality. Negative binomial regression to determine association between facility characteristics and availability of specialists in CHCs | Specialists (surgeon paediatrician obstetrician anaesthetist) | The Indian public health standards | NA | NA |
| Davey et al., 2019 [89] | Uttar Pradesh | Hospital, attached to Medical College, Meerut | Primary data | Cross sectional survey | 100 GNM qualified nurses | Goldberg and Hillier’s 28‑item scaled version of the General Health Questionnaire | Chi-square test | Nurses | INC guidelines | Exploration To Mid-Career | Employed |
| Dhusia et al., 2019 [90] | Mumbai | Four public sector hospitals | Primary data | Cross-sectional survey | 300 resident doctors | Copenhagen Burnout Inventory questionnaire | Descriptive statistics | Resident doctors | MCI guidelines | Not Mentioned | Employed |
| Nair et al., 2019 [91] | National level study | NA | Secondary data | Secondary research | NA | Websites of MCI, DCI, INC, Pharmacy Council of India, Indian Association of Physiotherapists, and the Ministry of Health and Family Welfare | Cross‑sectional survey | Doctors, surgeons, AYUSH doctors, dentist, nurses, pharmacists, and community health workers | NA | NA | Employed |
| Raphael et al., 2019 [92] | 42 LMIC,UMIC,HIC international survey | Web based survey | Primary data | Online survey | 1115 respondents | Web based survey | Kruskal Wallis test | Oncologist | NA | Mid-Career To Late Career | Employed |
| Sengar et al, 2019 [93] | 22 LMICs. | Web based survey | Primary data | Cross sectional survey | 82 medical oncologists | Online survey | Descriptive statistics, man Whitney u test, Chi square test | Medical oncologists | NM | Mid-Career | Employed |
| Srivastava et al., 2019 [94] | Delhi | Uttar Pradesh, Haryana, Delhi, and Madhya Pradesh | Primary data | Cross sectional survey | 240 doctors | Job satisfaction survey scale, 21-item QWL scale | Correlation, reliability, and mediator regression analysis | Doctors | NM | Exploration To Decline | Employed |
| Goel et al., 2019 [95] | Himachal Pradesh, Haryana, and Punjab) | Medical colleges | Primary data | Mixed method study (both qualitative and quantitative) | 90 medical students | Focus group guidelines with open-ended questions | Descriptive statistics, thematic analysis | Medical students | NA | Exploration | Employed |
| Ray et al., 2020 [96] | Maharashtra | Rural areas in selected districts of Maharashtra | Primary data | Cross sectional survey | 200 Asha workers | Job satisfaction scale | Descriptive statistics, chi square test | Asha workers | NA | Exploratory To Establishment | Employed |
| Bharadwaj et al., 2020 [97] | Uttar Pradesh | Institution based study | Primary data | Cross‑sectional survey | 210 medical practitioners | Structured questionnaire | Structural equation model to explore the significance of proposed model. | Physicians, surgeons, ophthalmologists, dentists, gynaecologists obstetrics, paediatricians, and junior residents | NA | Exploration To Late Career | Employed |
| Ranjith et al., 2020 [98] | National level | Dentists working in both public and private sector | Primary data | Cross‑sectional survey | 188 dentists and interns | Structured questionnaire | Partial least-squares technique for Structure Equation Modelling to analyse the effect of organizational commitment and work pressure on the job satisfaction of dentists. | Registered Indian dentists and interns | NA | Exploration To Mid-Career | Both employed and unemployed |
| Kumar et al., 2020 [99] | National level | Commentary | NA | NA | NA | NA | NA | Specialists including surgeons, obstetricians & gynaecologists, physicians, and paediatricians | NA | NA | NA |
| Tessy Treesa Jose & Sripathy M. Bhat, 2021 [100] | Mangalore | Selected medical college hospitals and government hospitals | Primary data | Cross sectional survey | 1040 nurses | The Minnesota Satisfaction Questionnaire (MSQ)-Short form 1977 | Descriptive statistics, Kruskal Wallis, Mann-Whitney U test | Nurses | NA | NA | Employed |
| Kinariwala et al., 2021 [101] | Gujarat | Institution based study | Primary data | A cross-sectional, online survey | 403 Indian dentists | Structured questionnaire | Descriptive statistics | Dentists | NA | NM | Employed |
| Nair et al., 2022 [102] | 5 Indian States including Madhya Pradesh, Gujarat, Odisha, Tamil Nadu and Andhra Pradesh and Telangana | CHCs and PHCs in rural areas | Secondary data | Secondary research | NA |  | Retrospective analysis of existing facility data and modelling techniques. Kruskal–Wallis test to measure inter-state differences | PHC-nurses, PHC-doctors, CHC-nurses, CHC-GDMOs, CHC-physicians, CHC-surgeons, CHC-OBG, and CHC-paediatricians. | NA | NA | Employed |

NA: Not Applicable; MCI: Medical Council of India; NR: Not Reported; CHC: Community Health Centre; NM: Not mentioned; INC: Indian Nursing Council; CGHS: Central Government Health Scheme; IPHS: Indian Public Health Standards; OBG: Obstetrics and gynaecology; MO: Medical Officer; WISN: Workload indicators of staffing need; PHC: Public Health Centre; AP: Andhra Pradesh; BAMS: Bachelor of Ayurvedic Medicine and Surgery; BHMS: Bachelor of Homeopathic Medicine and Surgery; UG: Under graduates; AYUSH: Ayurveda, Yoga and Naturopathy, Unani, Siddha and Homeopathy; WHOQOL- World Health Organization Quality of Life; DCI: Dental Council of India; QWL: Quality of Worklife; ANMS: Auxiliary Nurse and Midwife; DHS: Directorate of Health Services; CDMO: Contract Development and Manufacturing Organization; IPHA: Indian Public Health Association; NICU: Neonatal intensive care unit; MBBS: Bachelor of Medicine, Bachelor of Surgery; ANOVA: Analysis of Variance; ASHA: Accredited Social Health Activist; CDSCO: Central and State-level Drug Regulatory Agencies; FDA: Food and Drug Administration; PNDP: Private non degree practitioners; FIP: International Pharmaceutical Federation; PCI: Pharmacy Council of India ; WHO: World Health Organization; LMIC: Low and Low Middle Income Countries; HIC’s: High Income Countries; UMIC: Upper Middle Income Country; GLOBOCAN: Global Cancer Observatory; DLHS: District Level Household & Facility Survey; GNM: General Nursing and Midwifery

**Supplementary Table 3: Outcomes of the selected studies**

| **Author and Year** | **Reason for shortage** | **Results** | **Conclusion** |
| --- | --- | --- | --- |
| Zeighami et al., 1978 [16] | Financial issues and lack of professional growth | Higher pay and the ambition to eventually work in a Western nation were the most frequently cited justifications for accepting the positions. | Financial incentives with better career opportunities for professional growth are required to prevent migration. |
| Goyal et al., 1979 [17] | Uneven distribution of doctors in health centres | Even if the health centres are located in the least desirable areas for doctors, adding more doctors to those locations may boost the effectiveness of the health services even when the work load (number of patients per day) is higher. | Based on the medical and health needs of the region the health centre serves, the number of doctors assigned to the facility must be determined. |
| Astor et al., 2005 [18] | Migration to other countries | Desire for higher salary (90.8%), easier access to advanced technologies (74.1%), stability and security (72.5%), and better prospects for one's children (78%) are the key factors for migration. | There aren't enough physicians to address the population's healthcare needs in their individual nations and to develop the healthcare infrastructure in those countries as a result of physician migration. |
| Bhat et al., 2005 [19] | Absence of professional development opportunities, unfair training opportunities, hiring and promotion process decrease the commitment of employees | Providing opportunities for CME and professional development, supporting regional training programmes, presenting chances for advancement, fairness in training opportunities, hiring, performance reviews, and in promotion processes help in increasing commitment of employees | In order to acquire the necessary service capability in the health sector, structural arrangements of the sector and developmental HR practises are the main intervention tools that are required. |
| Mullan, 2005 [20] | Better salary and job security in Developed countries | India (59,523), the Philippines (18,303), and Pakistan are the countries that have sent the most medical personnel to recipients (12,813) | The recipient countries receive the most doctors in absolute terms from India and the Indian subcontinent, but the relative draw on countries, as indicated by the emigration factor, is actually greater for sub-Saharan Africa and is particularly prominent for Caribbean states. |
| Thomas, 2006 [21] | Nurses in the private sector, as well as those from certain linguistic and religious groups, were found to be especially vulnerable to migration. | In addition to economic concerns, it was found that important variables driving Indian nurses' overseas migration were discontent with working conditions and displeasure with current social views regarding nurses. | Health policymakers in India must seriously consider the growing migration of nurses to foreign countries. While such migration results in the inflow of foreign exchange, it also results in the loss of medical personnel who are critical to the achievement of national goals. |
| De Costa et al., 2007 [22] | Migration and brain drain, the difference between rural and urban health indicators reflects the difficulty in obtaining care due to the scarcity of skilled providers in rural areas, which is exacerbated by inadequate infrastructure there and high poverty rates. | 75.6% doctors work in the private sector. 80% of private physicians practise in cities. The private sector employs 72.1% of all qualified paramedical professionals, primarily in rural areas. There are 41 qualified doctors for every 100,000 people, 120 doctors per 100,000 people in urban MPs, and 12 doctors per 100,000 people in rural MPs. | Overall, there is a shortage of 15,000 doctors. Every year, the province graduates between 800 and 1000 certified doctors. It would take 15 years to make up the current shortfall if population growth and health workforce exits were not taken into account. |
| Katrak, 2008 [23] | The need for medical care is greater and access is more difficult in rural areas, which results in a highly noticeable relative shortage there. | The performance of the unqualified doctors would need to be at least 25% to 30% of that of the qualified doctors in order for the quality-adjusted number of practitioners to be equal in rural and urban areas. | There are far more unqualified practitioners in rural regions of the Ujjain district than there are doctors who are relatively more qualified. |
| Alonso et al., 2009 [24] | Migration of Indian nurses to United Kingdom for different reasons | Financial reasons, social and professional ambitions, religious and gender related issues, family support and assistance from migrant network are the key reasons for migration. | The social and cultural contexts in which nurses reside have a significant impact on their decision to immigrate along with economic reasons. |
| Bhandari et al., 2010 [25] | Employers pay more focus on intrinsic job factors that affect doctors' job satisfaction, such as autonomy, acknowledgement of work, level of responsibility, and variety in work. | Job satisfaction levels were low in all categories of respondents | Physical Work Conditions, autonomy, unhealthy relations with colleagues, boss and management, salary are the key factors affecting job satisfaction |
| Bhattacharya et al., 2011 [26] | Work monotony, compensation and benefits, work-life balance, a sense of accomplishment, a heavy workload , the need for automation and technology advancement, brain drain and breaks from work are the six causes of attrition. | Gender, age, marital status, the type of employment, work history, and pay, high work load, a lack of social benefits, and a low pay structure were found to be significant factors in the reasons people change jobs. | At the start of the project, careful planning for human resources should be done, along with a strategy for their recruitment, training, and deployment. These are crucial considerations from project planning to implementation. |
| Sodani et al., 2011 [27] | NA | For doctors, surgeons, OBGs, paediatricians, anaesthesiologists, and MOs, there was a disparity of 9, 8, 8, 9, and 12, or 46%. For the positions of nurse, technician, pharmacist, and radiographer, respectively, gaps of 42, 27, 13, and 13% were detected | It was discovered that infrastructure facilities were available in almost all CHCs, but there was a shortage of manpower, particularly specialists. |
| Aggarwal et al., 2012 [28] | Dissatisfaction due to stressful life, difficult studies, brain drain because of better income and status | 64.5% were happy to be studying dentistry professionally. Interns were most discontented. 66.9% plan to travel abroad for career advancement. | To increase student happiness, the admissions procedure for dental students’ needs to be thoroughly reviewed. |
| Hagopian et al., 2012 [29] | Migration of Indian HRH to nations with higher incomes | The health workforce supply to be increased by 80 nurse midwives, 15 additional nurses, and 43 additional physicians in order to adequately serve the study population. | Delivering the services promised by the NRHM will need a substantial influx of additional health professionals. |
| Kadam et al., 2012 [30] | The main causes of dissatisfaction included existing promotional opportunities following the conditions of rural service, a lack of physical infrastructure, and a lack of educational opportunities for the children of health personnel. | Government allopathic doctors, lab technicians, and staff nurses make up 13000, 40000, and 15000 of the population, respectively. | The majority of health professionals considered a strong personal commitment to helping others, physical infrastructure, training opportunities, senior support, good schooling for their children, and promotion opportunities after a certain number of years of working in rural areas to be important factors for continuing to work in these settings. |
| Murthy et al., 2012 [31] | Less salary, and poor infrastructure leads to dissatisfaction | Political meddling and a lack of high-quality educational resources for kids in rural areas were major obstacles. One of the main requirements for doctors working in rural locations is security. Other important requirements are living conditions, connectivity, and proximity to family. | This package would need to include a pay raise, greater post-graduate education prospects, better-equipped and stocked health facilities, better housing conditions, and transparent transfer regulations. |
| Rao, 2012 [32] | NA | The most effective factors influencing students' and in-service professionals' acceptance of rural posts are higher salaries, strong facility infrastructure, and ease of enrollment in higher education programmes. Combining these incentives can significantly boost rural hiring | It seems difficult in India to encourage medical graduates to work in rural areas. This can be somewhat remedied by making entry to specialised training simpler and hiring students from rural areas. Contrarily, nursing students and in-service nurses are far more open to rewards for using rural services. |
| Rathore et al., 2012 [33] | NA | The findings demonstrated that female nurses faced numerous issues with their health and well-being, exhaustion, and social and household circumstances. | The nurses had to make adaptations owing to shift work while still attending to the needs of the family, especially the children. |
| Saha et al., 2012 [34] | NA | Doctors think that the public sector offers greater job security than the private sector. In the government sector, factors including the working environment, accountability, career advancement, and compensation are not up to par. | The choice to practise in rural areas is the result of the complex interaction of many factors, including a person's background, the infrastructure of the services they provide, and the policies and procedures governing their use of human resources, including opportunities for career advancement, compensation, and autonomy. |
| Saini et al., 2012 [35] | NA | Infrastructure and salary were perceived as potential barriers to a rural health career. | Initiatives to strengthen rural areas, such as bettering transportation options and lowering health risks, would significantly lessen the need for practitioners in those areas. It is also necessary to develop training programmes to raise the standard of practitioners in rural areas. |
| Tiwari et al., 2012 [36] | When it came to transfers, it was found that medical staff weren't being handled fairly. The doctors acknowledged a moderate level of discontent with their pay. Only 11.25% of doctors who joined the state workforce 5 to 10 years ago received promotions. | The posting, perception of specialisation, hierarchical satisfaction, quality of facilities and services, and human resource policy are the drivers of worker availability. | NA |
| Rao et al., 2012 [37] | Inequitable distribution of HRH | The number of doctors, nurses, and midwives in India is only one-fourth of the World Health Organization guideline of 2.3/1000 people, indicating a severe general deficit of health professionals. 37% doctors especially in rural areas lack credentials. | Having more skilled health workers in underserved areas and having a more effective skill mix are two of India's most urgent human resource concerns. |
| Abraham et al. , 2013 [10] | Job dissatisfaction due to stress, burnout and poor QOL may result in turnover | Nurse educators needed assistance more frequently and experienced higher levels of burnout. The QOL of staff nurses was lower than nurse educators | Ineffective management of nurses may cause major stress reactions, which can further contribute to job discontent, burnout, and poor QOL. |
| Hazarika, 2013 [38] | Uneven distribution of Health Workforce | There is a severe lack of healthcare workers in some states. Doctor and dentist distributional disparities are the most pronounced and have a substantial impact on health outcomes. | Although the number of health professionals produced has increased significantly in recent years, the disparities in their distribution continue to be a problem. |
| Ramesh et al., 2013 [39] | Salary, financial perks, and pay fairness were all very significant factors and the absence of these benefits may have an adverse effect on dedication, satisfaction, and performance. | Payment, including salary and financial incentives, was a significant contributor to nurses' dissatisfaction, which in turn impacted their QWL. | The recent analysis identified the nurse workforce shortage as the primary issue. The existing nurses are overworked as a result of the shortage. |
| Pandey et al., 2013 [40] | Inequitable distribution of HRH | To provide the volume of healthcare services in its annual statistics in accordance with the professional standards for these services, according to WISN calculations, District Hospital, where the study was conducted, needs 16 laboratory technicians; i.e. there is a shortage of 10 technicians. | Due to a manpower shortfall, the laboratory technicians at the District Hospital where the study was done are continuously under pressure to keep up with the workload. |
| Kumar et al., 2013 [41] | Irregular vacancy planning | Regular vacancy planning is not done at the district level. The district health societies hire only contractual staff at the district level. Policymakers believe that contractual staff performs relatively better under pressure, and that the 'hire and fire' principle works better for contractual staff. | Human resource management necessitates the immediate attention of policymakers in order to create an organisational culture that allows for the best use of human resource potential. |
| Ramani et al., 2013 [42] | NA | Salary, working circumstances, children's education, living conditions, and safety are all non-financial elements that have an impact on health workers' job decisions. | India's rural retention programmes have mainly focused on the allopathic cadre. Rewarding rural service for the Ayurvedic and nursing professions is easier than it is for the allopathic profession. Therefore, it becomes sense to improve rural incentive programmes for these two cadres as well. |
| Rao et al., 2013 [43] | NA | Jobs in urban areas with urban amenities had a higher chance of being accepted. Increased pay had a small effect on doctors, but a large impact on nurses' acceptance of rural jobs. | The policy of reserving seats for specialised training in exchange for rural service had a significant impact on job acceptance among doctors, nurses, and nursing students. |
| Sodani et al., 2013 [44] | NA | At CHCs, there was a shortage of manpower, particularly specialists, whereas at 24*7 PHCs, there was a shortage of laboratory technicians and pharmacists. | Significant deficiencies were discovered in the CHCs and 24X7 PHCs studied in the Bharatpur district, and health facilities can be improved with additional inputs such as recruiting staff. |
| Purohit et al., 2014 [45] | Inadequate salary, no opportunities for promotion, no respect and non-recognition leads to dissatisfaction among Mos. | Interesting work, Respect and recognition, Adequate salary, Sound organizational policies and Opportunities for promotion are the key factors for MO’s. | The most vital factor affecting motivation was job security, followed by fascinating work, respect, and recognition. |
| Garner et al., 2014 [46] | Insufficient remuneration to cover basic requirements, conflicts between work and personal obligations, and safety and security concerns when travelling to and from work lead to dissatisfaction among nurses. | The perceived difficulties with a hierarchical structure and those caused by the shortage of nurses, safety, pay, and balance, Migration, work pressure, physical demand, inconsistency between theory and practise, and knowledge affect nursing workforce capacity. | In India, nursing as a profession is still in its infancy when compared to international standards. |
| Gupta et al., 2014 [47] | Better salary and job security in government sector | Nursing professionals working for the government reported higher job and pay satisfaction. | In comparison to private nurses, government nurses are shown to have higher levels of job satisfaction. |
| Halappa et al., 2014 [48] | Uneven distribution of Dental Health Workforce and Immigration & migration of dentists, changing disease pattern & treatment | There are 0.088% dental health professionals for every 1000 people in India. In India, there are disparities in the distribution of dentists. Some states, like Karnataka, Maharashtra, and Tamil Nādu, have a severe deficit of dental health professionals, but other cities, like Jharkhand, Rajasthan, and Uttaranchal, have an overabundance of dentists. | Despite the fact that the number of dental colleges has increased significantly in recent years, the distributional challenges that existed before still exist. In the battle to increase the number of dentists overall, an unequal distribution of dentists who are properly qualified, motivated, and supported creates a false sense of job saturation that discourages young people from choosing dentistry as a vocation, which is alarming. |
| Kumar et al., 2014 [49] | Discrimination and employee demotivation have been brought about by disparities in pay, leave policies, and other perks inside the firm. | Regularly updated HR planning is not performed, and as a result, actual HR requirements are not calculated, creating a backlog of shortages. | Despite having a sizable staff, the state health department lacks a professional HR department to provide guidance on numerous HR duties. Ad hoc employees are not provided the same benefits as regular employees who work for extended periods of time. |
| Bhatia et al., 2014 [50] | NA | The most chosen motive was "job stability," followed by "sufficient income," and "local political involvement" was the least preferred. For ad hoc MOs, "sufficient income" and "interesting work" were the two most crucial motivating elements, but for bonded MOs, "enough salary" and "opportunity for promotion" were the two most crucial motivating factors. | It iss crucial for health departments and systems to focus on creating plans that take both extrinsic and internal elements into account. In addition to paying MOs larger wages and other financial perks, more attention should be paid to ensuring their jobs are secure. |
| Sodani et al., 2014 [51] | NA | There was a shortage of human resources, particularly laboratory technicians and pharmacists. It was also discovered that none of the twenty-four seven PHCs have a fully equipped new-born corner. | According to the IPHS, the availability of human resources, infrastructure, and facilities for new-born care services at the 24 7 PHCs was insufficient. Priority must be given to strengthening OT, investigative facilities, and communication facilities at 24 7 PHCs. |
| Singh et al., 2014 [52] | The number of health professionals at HSC is strongly tied to the building's ownership, the presence of a phone, water, working restroom, regular energy supply, and the distance from the district office and closest bus stop. | Instead of the IPHS standard of four health workers, an HSC in India typically has two. ANMs and male health workers are absent from about 3.8% and 51.6% of HSCs, respectively. | When separated into "within state" and "between state" inequality, within state inequality makes up a significant portion (70%) of the overall inter-HSC inequality. |
| Ahmad et al., 2015 [53] | Dissatisfaction due to increased workload and teaching responsibilities | 66.3% were satisfied with the job, while 57.9% were satisfied with their workload. | Academics in the private sector were less satisfied with their workload and were more burdened by teaching responsibilities. |
| Amin et al., 2015 [54] | Job dissatisfaction due to stress, burnout and secondary traumatic may result in turnover | 70.5% nurses were found to have moderate to high levels of stress. 19.4% reported high compassion satisfaction, 23.3% reported high secondary traumatic stress and burnout. | Domains of work-related quality of life are associated with perceived stress. |
| Bhattacharya et al., 2015 [55] | Support from the organisation, its culture, values, and beliefs, has an impact on how united a healthcare professional feels with the group impacting employee retention. | The respondents' total satisfaction level was 51%. Availability and use of information technology were the key factor affecting job satisfaction and retention. Compared to nurses and administrators, doctors expressed greater dissatisfaction. | The healthcare industry has the greatest rates of attrition, making it difficult to retain vital human resources. |
| Garner et al, 2015 [56] | Lack of rewards and recognition may lead to dissatisfaction. | Two key themes emerged were perceived intrinsic rewards including service, blessings, holistic care, personal pleasure, noble professions, and illuminating lives and lifelong rewards including education, job security, importance, and variety. | One tactic to change societal perceptions of nursing in India and to increase the number of nurses recruited in a nation with a severe nursing shortage is to emphasise the benefits of nursing. |
| Nallala et al., 2015 [57] | Less salary, and poor infrastructure leads to dissatisfaction for working in rural areas. | Only 17% of students indicated a long-term interest in working in rural regions after graduation. 9.2% were prepared to work in a rural location after completing their PG. Nearly 30% of students indicated their readiness to work in rural areas after graduation. | The problem may not be solved by expanding the number of locations or building new medical institutions because students who get public funding for their education are unmotivated to work in remote areas. |
| Yashika Negi & Rajni Bagga, 2015 [58] | Dissatisfaction with work related factors leads to turnover. | The amount of night shifts, pressures placed on patient attendants, patient deaths or critical conditions, conflicts at work, study leave, and the category of employment were all work profile factors that were linked to burnout. | Burnout was found to be significantly correlated with specific organisational and work profile characteristics. |
| Saxena et al., 2015 [59] | NA | Public hospital employees were more satisfied with their recruitment and selection process, less committed to their organisation, and had lower levels of occupational stress than private hospital employees. | Healthcare workers who were pleased with the recruitment, selection, training, and development process were also pleased with their job and career. |
| Zodpey et al., 2015 [60] | Unfair recruitment policies and training opportunities | Progress towards universal health care would be challenging given the current shortages and unequal production and recruitment distribution, taking into consideration the HRH requirements for a typical Indian region. | Government should to create a thorough national health policy for health human resources and to secure funds for the training of the health workers. |
| Ahmad et al., 2016 [61] | dissatisfaction due to unsuitable government policies, lack of management in the pharmaceutical council, a high workload and low income. | 17.5% pharmacists were satisfied with their profession. 14.58% expressed satisfaction with the regulatory agencies' contribution to the growth of their profession. | Inappropriate government policies followed by Low income, a lack of management in the pharmaceutical council and high workload. |
| Purohit et al., 2016 [62] | Lack of motivation | Intrinsic motivation is the most important factor than extrinsic motivation | This study concludes that intrinsic and extrinsic motivation variables are crucial for healthcare personnel to increase their desire to perform better at work. |
| Purohit et al., 2016 [63] | Employment perks and job security closely associated with recruitment practises and procedures have an impact on the state's scarcity of MOs and high turnover. | Incredibly complex, non-transparent and dispersed Recruitment Rules, slow and extremely erratic recruitment process, health department's protracted delivery of wage benefits and service regularisation, unequal opportunities with regard to job stability, no wage benefits, and non-acknowledgement of prior work experience are key factors influencing the attraction of MO’s. | In terms of strategic HRM, better doctor availability, distribution, and management depend heavily on attracting, keeping, and managing HRH effectively. |
| Pandey et al., 2016 [64] | Low levels of surface-level emotional labour leads to high burnout and low levels of job satisfaction. | The negative correlation between surface-level and deep-level emotional labour techniques was a significant finding. Additionally, research shows that high levels of emotional labour on the surface are linked to decreased burnout and higher levels of job satisfaction. | Soft emotional management skills are crucial for community healthcare and must be developed at the grassroots level. |
| Kadam et al., 2016 [65] | Frustration develops when doctors are kept on the periphery for an extended period of time without being promoted. | In the state, a quarter of the doctor positions were unfilled at all institutional levels. The majority of doctors who were interviewed had little idea about the placement, transfer, or promotion policies that are currently in effect. | The government faces a challenge in deploying doctors in an adequate and acceptable manner since it must balance employee desires with organisational requirements. |
| Kadam et al., 2016 [66] | The causes for the ongoing inability to meet the required criteria included recruitment delays, legal obstacles, a delayed recruitment procedure, and a lack of career options. | In Maharashtra, 55% of the authorised drug inspector positions were unfilled in 2009–2010. Based on the recommendations of the Mashelkar Committee, this led to an 83% shortage. Less than 25% of the necessary inspections of the production and sales units were carried out. | The proposed workforce planning standards of the Mashelkar Committee are not met by the Maharashtra Food and Drug Administration (FDA). To bring about the necessary reforms, legislation, political backing, and operational assistance are required. |
| Purohit et al., 2016 [67] | NA | There is no official published or written first posting policy in the state. The state has various unwritten customary customs, such as deploying the Medical Officers in their home regions, in the lack of any explicit policies. | There must be clearly defined initial posting-related policies that show equity and consideration towards Medical Officers in placement-related matters if long-term solutions to the availability and distribution of Medical Officers in the state are to be explored. |
| Ranga et al, 2016 [68] | NA | 25% of all active healthcare professionals across the research areas are private non-degree practitioners. The primary workforce for OPD care is made up of PNDPs, who operate within a 2–5 km radius. | When all healthcare providers are taken into account, PNDPs offer the best possible accessibility to OPD care. Remote places will be most impacted if the PNDPs are eliminated from the scenario, leaving them with essentially no OPD treatment. |
| Rao et al., 2016 [69] | Unequitable distribution of health workers in India. | Overall low numbers of qualified health workers, the prevalence of unqualified health workers, especially in rural areas, and the notable differences in qualified health worker distribution between urban and rural areas need to be examined. | There is substantial unequitable distribution of health workers in India. |
| Ambadekar et al., 2017 [70] | Varying expectations and attribute availability leads to dissatisfaction which may affect employee turnover. | 75% of the study participants believed that "excellent working relationships," "training opportunities," and being "respected and trusted by clients" were the more important aspects" of a profession. | Work overload, lack of opportunities for career advancement and training, and social security issues are the key reasons affecting employee satisfaction. |
| George et al., 2017 [71] | Lack of motivation at community centres | Community health workers were demotivated by negative experiences in the community and at health centres, limitations in the local health system as a result of the demand that the community health workers produced, and low compensation, which even led some of them to quit their professions. | Programs for community health workers that prioritise improving the technical proficiency of their employees may not pay enough attention to the elements that encourage or deter these workers. |
| George et al., 2017 [72] | Migration of Indian HRH to nations with higher incomes | Compared to India, nurses in the USA might make up to 82.7% more money while in Canada and the UAE, they are better off by up to 28 and 20%, respectively. Only nurses working in the UK may have a considerably worse situation than nurses in India. | It is difficult for India to retain skilled medical personnel due to the movement of Indian HRH to nations with higher incomes, which affects government efforts to make healthcare more accessible throughout the nation. |
| Janakiram et al., 2017 [73] | Lack of career advancement and less opportunities for students to apply their whole set of skills to the practise of public health. | Almost half of the participants thought they had yet to succeed in one's current career as a prominent figure. Only 46.9% of respondents said there had been progress in the dental health occupation as career | Given that the jobs are in a falling stage, there has been some hesitation or pessimism over the specialty's future. |
| Pandey et al., 2017 [74] | lacking essential amenities, not having access to drugs and equipment, having a bad infrastructure, working long hours without pay, and being physically abused by patients' relatives in government leads to migration of doctors to private hospitals | 4432 paediatricians are needed to fill the present vacancies, and if they are added at the average rate of 29 each year, it will take 152 years to do so. 4479 physicians are needed at the CHC level, and at the current pace of 3.4 physicians per year, it will take 1317 years to fill the shortage. | In India, there is a complete separation between the medical education system and the healthcare system. |
| Patil et al., 2017 [75] | Absence of recruitment of staff | 35% of SCs provided services to the population in accordance with the norms. About 33% of SCs lacked any structures. The lack of staff housing in half of the SCs was a weakness worth addressing in the current study. | The district's SC level does not adhere to IPHS norms. To ensure that SCs operate effectively, all SCs should hire workers, in particular to fill the male health worker position. |
| Pudpong et al., 2017 [76] | NA | The majority of nursing students in five countries came from rural areas. Students in India (67.1%) and Thailand (65.1%) were more enthusiastic about working in rural areas. Students in Bangladesh (78.8%) and India (62.6%) thought their schools prepared them well for rural work and inspired them to do so. | Nursing students who were raised in rural areas and recruited in rural areas had more positive attitudes toward rural areas and were more likely to choose to work in rural areas after graduation. |
| Rajbangshi et al., 2017 [77] | NA | Rural background and community attachment were found to be strongly related to health workers' decision to join rural service, regardless of cadre or contract. However, health-systems factors such as poor working and living conditions, low pay and incentives, and a lack of professional growth and recognition hampered this aspiration. | Crisis in recruiting and retaining rural health workers will persist until and unless health systems address the core basic needs of rural health workers, which are related to health-sector policies. |
| Walton-Roberts et al., 2017 [78] | NA | There are shortages of health workers in some regions of India and in some speciality fields, but it is challenging to assess the scope and type of such shortages due to a dearth of research and health statistics. | Rather than being a direct outcome of health worker international migration, India's scarcity of trained health professionals should be considered in relation to domestic policy on training, recruitment, and retention. |
| Motkuri et al., 2017 [79] | Regulatory issues | In India, there is a severe scarcity of human resources for health care services, particularly skilled health professionals and labourers, which is quite concerning. The burden of insufficient health care services has fallen disproportionately on rural populations. | To monitor health outcomes and inform the state policy-making body to adjust course for the better, India needs a comprehensive information system on many aspects of health care. |
| Bates et al., 2018 [80] | workforce expansions are not at pace with Population growth and changing dynamics of regional disease burden | The number of pharmacists in Eastern Mediterranean and Africa between 2006 and 2012 has increased, but the World Bank's low- and lower-middle income countries and territories still have a lower pharmacist density than high- and upper-middle income countries and territories. | All nations and areas are experiencing a rise in employment, which is a positive development for better access to and availability of pharmaceutical expertise. |
| Saikia, 2018 [81] | Most of the NRHM's high-focus states have nurse and midwife shortages that are worse than the national average. Along with a significant number of the necessary positions across states/UTs remaining unfilled, there is also the grave issue of vacancies in sanctioned offices at all levels. | There are 2.78 million registered nurses and midwives, up from 1.23 million in 2000, 1.48 million in 2005, and 2.78 million in 2016. There were 12.1, 13.5, and 21.9 nurses and midwives per 10,000 persons in 2000, 2005, and 2016, respectively. | Although the number of nurses in the rural public health sector has gradually increased from 2005 to 2017, both in absolute terms and in relation to the population being serviced, the sector is still experiencing a severe shortage of nurses. In comparison to international norms, nurse densities and the doctor-to-nurse ratio are appallingly low, and these numbers vary greatly between states and union territories. |
| Fundytus et al., 2018 [82] | Oncologists in LMICs see significantly more patients, work longer days, are on call more frequently, and have less vacation time than their global colleagues, less likely to work in the public sector and have less access to complementary cancer services. | In comparison to UMICs and HICs, the annual case volume in LMICs was significantly higher. The median number of working days per week in LMICs, UMICs, and HICs was 6, 5, and 5, respectively. Pakistan (median consults, 950; 73% > 500 consults), India (median visits, 475; 43% > 500), and Turkey (median consults, 475; 27% > 500) had the largest annual case loads per oncologist. | Medical oncology case volumes and clinical workload vary widely across the world; this is particularly noticeable in LMICs, where severe shortages occur. |
| Mathew, 2018 [83] | NA | In none of the nations in Europe or the Americas had the mortality-to-incidence ratio > 70%. In seven South American countries (100%) and 13 European countries (42%) respectively, the mortality-to-incidence ratio exceeded 50%. | The prevalence of cancer and the lack of human resources are strongly correlated with the economic and social development status of a nation. |
| Oda et al., 2018 [84] | The main driver of nurses moving abroad is economic considerations. | Over 600,000 Indian nurses are employed abroad, and there is currently an estimated 2.4 million nursing shortage in India. | Low pay and unfavourable working conditions, particularly in the private sector, are the main drivers of Indian nurses leaving India for other countries. |
| Osman et al., 2018 [85] | The shortage of doctors is caused by a limited capacity for physician training and skilled worker immigration within and between areas. | The number of nephrologists per million people (PMP) worldwide was 8.83; in high-income nations, it was 28.52; in low-income countries, it was 0.31. | There are shortages of all nephrology care providers worldwide including India, and significant variance in the density of nephrologists and nephrology trainees. The gap was especially pronounced in low-income communities. |
| Tiwari et al., 2018 [86] | NA | 3,463 qualified health management professionals were available in total in 2017. India would require 39,774 health management specialists in 2017 to reach the normative threshold of 2.97 health managers per 100,000 people. By 2030, there would be a demand for about 44,936 health management specialists to maintain the normative requirement of 2.97 health managers per 100,000 people. | A competency framework that is specifically specified, standardised, and adapted to the Indian context is needed to raise the output quality. |
| Motkuri et al., 2018 [87] | Inequitable distribution of HRH in urban and rural areas | Throughout India and across states, there was an increase in the density of health workers between 1991 and 2011, especially in the last ten years (2001-11). The density of health professionals in rural areas is still 1/3rd that of urban areas. | Introduction of ASHA employees since 2005 with the implementation of NRHM has greatly contributed to the increase in rural health worker density. |
| Singh, 2019 [88] | 1. Less number of PG seats in some courses. 2. Unavailability of residences, water, electricity, location of a CHC and distance to district headquarters. 3. Weak financial and non-financial incentive system for working in rural areas. 4. lack of a central HRH database | 80.7% of the required number of specialists are not available. 62.1% of CHCs are operating without a specialist. | Distribution of experts among CHCs has been progressively more uneven. |
| Davey et al., 2019 [89] | Reasons of dissatisfaction include Male patients' unfriendly behaviour, unsanitary hostel conditions, placement in high-volume areas with increased workload, and low pay. | Mild to moderate/severe levels of job-related stress followed by poor treatment from male patients, the lack of a separate washroom, postings in busy departments with increased duty, and low pay were the most frequent concerns. | Monitoring and evaluating stress levels and job satisfaction over time is necessary; it cannot be done in a single step. |
| Dhusia et al., 2019 [90] | Burnout syndrome including physical, emotional, and psychological effects lessen their capacity to successfully care for patients and the community. | The average number of hours worked per week was 88. 56.66% of participants displayed burnout-related ratings. There was personal burnout in around 66.67%, work-related burnout in about 57.14%, and client-related burnout in 16.67% of respondents. | The high rate of burnout syndrome among resident doctors in public sector hospitals is worrisome since it not only has a negative impact on the physical and mental health of medical professionals but also lowers their motivation and productivity at work. |
| Nair et al., 2019 [91] | Some of the major obstacles to the retention of health professionals in rural areas are inadequate financial compensation, a lack of effective clinical infrastructure, and organisational policies and management. | India suffers a severe shortage of health workers, especially doctors and specialists, in the rural areas of several states, as evidenced by the open posts in the primary health system, despite a vast growth in the supply of health workers. | In order to overcome health system obstacles and accomplish rural recruitment and retention across multiple cadres in states, focused attention and long-term political commitments are needed. |
| Raphael et al., 2019 [92] | Oncologists with low job satisfaction are more likely to work in the public system and have less access to supporting services such as radiotherapy, palliative care, and chemotherapy pharmacists | Lack of access to on-site radiotherapy, palliative care, and chemotherapy pharmacists as well as increasing weekly hours worked, declining annual weeks of paid vacation, being on call every night, higher clinic volumes, and being on call every night were all linked to low job satisfaction. | It is costly to incentivize doctors to serve in rural areas. A broader strategy of significant salary increases with improved living, working, and educational incentives is required. |
| Sengar et al, 2019 [93] | NA | Indian MOs have higher clinical volumes and workload than MOs in other LMICs and substantially higher workload than MOs in high‑income countries. | NA |
| Srivastava et al., 2019 [94] | NA | There is a substantial negative association between job burnout and job satisfaction, a significant positive relationship between job satisfaction and QWL, and a significant negative relationship between job burnout and QWL. | As burnout cannot be totally eliminated from the workplace of doctors and since not all job needs can be condensed, hospital administration must address numerous essential job demands that foreshadow burnout by developing a variety of therapies. |
| Goel et al., 2019 [95] | NA | Disappointing factors were divided into two categories: a professional environment that isn't challenging and an imbalance between financial benefits and social disadvantages. | India must provide the most basic medical infrastructure in rural areas. It is necessary to review the medical curriculum to give the needs of the entire nation's population top priority. This includes early and ongoing clinical exposure to rural areas during medical training and equitable distribution of health services among urban centres and rural villages. |
| Ray et al., 2020 [96] | Working environment, rewards, workload and leave policy, | 58.5% ASHA personnel were not happy with their jobs. 15.5% of respondents reported having very high levels of job satisfaction, compared to 26% who reported moderate levels. | Significant portion of ASHA personnel were unhappy with their working environment, rewards, workload, leave policy, and with their position. |
| Bharadwaj et al., 2020 [97] | Occupational stress is a significant predictor of job dissatisfaction and usually result in turnover. | The importance of the family-work balance was highlighted most on engagement and least on medical specialists' satisfaction. | The organization's structure is the most important indicator of medical professionals' happiness, followed by environmental factors, job demands, and family-work balance. |
| Ranjith et al., 2020 [98] | Job satisfaction and level of income satisfaction are the predictors of turnover intention | Personal requirements for life quality (37%) lack of recognition (29%) and comparison of pay with peers (29%), plans to emigrate (22%), and unhappiness with compensation (22%) were the variables that had the greatest impact on respondents' intentions to leave their jobs. | The likelihood that the respondent will leave the organisation will increase with decreased organisational commitment, job satisfaction, level of financial satisfaction, and age. |
| Kumar et al., 2020 [99] | Infrastructural issues and improper training | The overall shortfall of specialists employed at the CHC level is 81.9%. There is a scarcity of doctors (85.7%), surgeons (84.6%), obstetricians and gynaecologists (74.7%), and paediatricians (82.6%). | India's health care systems and services are still in the process of evolving, and they face issues with a lack of skilled workers, absenteeism, inadequate infrastructure, and care quality. |
| Tessy Treesa Jose & Sripathy M. Bhat, 2021 [100] | Insufficient remuneration and improper duty hours guidelines | Only 0.96% workers reported having a high level of job satisfaction, whereas 93.94% reported being unsatisfied with their jobs. | Nurses in many facilities are underpaid, which may have an effect on their job happiness. For nurses' daily duty hours, there is typically no guideline in most institutions. |
| Kinariwala et al., 2021 [101] | Unprecedented factors like pandemic or epidemic can have psychological toll on medical professionals | 35.7% of dentists were reluctant to start their post-pandemic practises. 35.5% of respondents were worried about rising treatment costs due to additional infection control measures, and 45.9% were worried about the possibility of catching SARS-CoV-2 infection from a patient. | Due to professional and/or medico-legal considerations, dental professionals in India generally appear underprepared to provide patient treatment during or after the COVID-19 outbreak. |
| Nair et al., 2022 [102] | For all HRH cadres, present sanctioning criteria do not align with WISN-based requirements, falling particularly short for nurses and specialised physicians. | Results demonstrated low concordance between WISN-based requirement and sanctioning, particularly for nurses and specialised doctors, and under-sanctioning in several states. | The existing sanctioning standards require an evidence-based update. Workload-based HRH deployment in rural regions will guarantee sufficient availability and equitable distribution, which are required to raise the general standard of rural healthcare. |

NA: Not applicable; HR: Human Resources; CME: Continuing Medical Education; CHC: Community Health Center; HRH: Human resources for health; NRHM: National Rural Health Mission; QOL: Quality of Life; QWL: Quality of Work life; PHC: Primary health care; MO: Medical Officer; OT: Operation theatre; IPHS: Indian Public Health Standards; OPD: Outpatient department; UAE: United Arab Emirates; UT: Union Territory; NRHM: National Rural Health Mission; ASHA: Accredited Social Health Activist; LMIC: Low and Low Middle Income Countries; HIC’s: High Income Countries; UMIC: Upper Middle Income Country; WISN: Workload indicators of staffing need; SARS-CoV-2: Severe Acute Respiratory Syndrome Coronavirus 2.

**Supplementary Table 4: Codes identified under each theme**

| **Themes** | **Codes** |
| --- | --- |
| Inadequate HRH Production | - Large Population size - Unequitable rural-urban HRH distribution - Demographic shift - Medical tourism - Limited PG seats |
| Job dissatisfaction | - Subpar facilities - Insufficient equipment - Underpaid staff - Few opportunities for professional growth |
| Brain Drain | - Desire for better pay - Better living - Unable to obtain employment. - Lack of advanced studies or training opportunities |
| Regulatory concerns | - Inadequate staffing and training regulations - Protracted wage benefits delivery - Protracted service regularisation - Unequal job stability opportunities - Non-acknowledgment of prior work experience - Complex and dispersed recruitment rules - Slow and erratic recruitment process |
| Lack of training, monitoring and evaluation | - Difficulty picking up new skills and information - inefficient monitoring and evaluation methods - Unmatched skills for healthcare demand |
| Regulatory issues | - Increase allocation of GDP to health - HRH cadres - Vacancy and appraisal system - Increase HRH - Public health facilities - HRH ratios as per country’s needs - Administrative policies |
